# Suppressing the OTUD7A/KDM5B/GABPA axis enhances the sensitivity of cisplatin through inducing ferroptosis in KRAS-mutant LUAD

**DOI:** 10.1038/s41419-025-08337-x

**Published:** 2025-12-20

**Authors:** Rujia Si, Ziyang Shen, Ying Sui, Yue Shi, Yihan Zhang, Bowen Hu, Xin Chen, Bing Feng, Miao Lin Zhu, Xiaofeng Sha, Ning Ding, Guoren Zhou, Feng Jiang, Cong Xu, Bo Shen

**Affiliations:** 1 Department of Oncology, The Affiliated Cancer Hospital of Nanjing Medical University, Jiangsu Cancer Hospital, Jiangsu Institute of Cancer Research, Nanjing, China; 2 Department of Medical Oncology, Hongze District People’s Hospital of Huai’an City, Huai’an, Jiangsu Province China; 3 Wuxi Huishan District People’s Hospital, Wuxi, China; 4 Department of Thoracic Surgery, Jiangsu Cancer Hospital, The Affiliated Cancer Hospital of Nanjing Medical University, Jiangsu Institute of Cancer Research, Nanjing, China

**Keywords:** Non-small-cell lung cancer, Ubiquitylation

## Abstract

KRAS-mutant lung adenocarcinoma (LUAD), due to its evolution of more complex antioxidant metabolic mechanisms, exhibits poorer sensitivity to conventional platinum-based drugs compared to other types of LUAD. Ferroptosis, as a means of inducing cell death in cancer therapy, shows unique features and potential therapeutic effects compared to the conventional form of apoptosis, which is frequently obstructed by drug resistance. In human KRAS-mutant LUAD cell lines and mouse models, we found that the deubiquitinase OTU deubiquitinase 7A (OTUD7A) precisely regulates the lysine demethylase 5B (KDM5B). Inhibition of KDM5B expression increases the H4K20me3 level, which in turn downregulates the expression of transcription factor GABPA associated with mitochondrial function, ultimately promoting the production of more Reactive Oxygen Species (ROS) by mitochondria and inducing ferroptosis. Additionally, in in vivo organoid models, cisplatin (CDDP) induced ferroptosis combined with GABPA inhibition demonstrated superior anticancer effects compared to conventional platinum-based drugs. This research identifies new targets and regulatory networks that hold promise for developing ferroptosis-based therapies for KRAS-mutant LUAD.

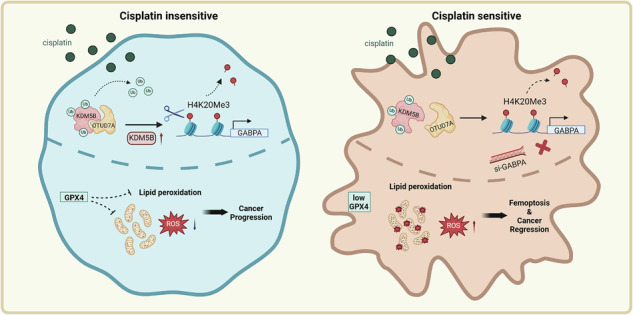

## Introduction

Lung cancer ranks first globally in terms of both incidence and mortality rates [1]. The KRAS target has instilled hope in past researchers to overcome this challenge [2]; however, the shallow active pocket of KRAS has resulted in weak drug binding affinity [3–6]. Therefore, it is crucial to investigate effective treatments for KRAS-mutated LUAD. Histone methylation is an essential mechanism of epigenetic regulation, with the loss of methylation at particular sites profoundly affecting tumor progression and therapeutic responses [7, 8]. For instance, in triple-negative breast cancer, the demethylation of H3K27me3 enhances chemotherapy resistance and promotes peritoneal metastasis, whereas the inhibition of H3K27me3 can prevent drug resistance and delay tumor recurrence [9, 10]. Similarly, in liver cancer, reports indicate that suppressing H3K9me3 methylation reduces the expression of the oncogene S100 Calcium Binding Protein A11 (S100A11), thereby inhibiting the progression of hepatocellular carcinoma [11]. KDM5B, also known as PLU-1 or JARID1B, has received attention after Zhang SM and colleagues confirmed its role in promoting cancer progression and immune evasion by tumor cells [12]. However, the exact mechanism of its oncogenic role has mainly remained unknown [12–17].

Deubiquitination is crucial for stabilizing proteins and regulating their levels, and plays a significant role in KDM5B expression [14]. An imbalance in the ubiquitination system in tumor cells enhances malignant behaviors, including resistance to growth inhibition, metabolic reprogramming, and increased phenotypic plasticity [18–22].

This study reports for the first time that in KRAS-mutant LUAD, OTUD7A deubiquitinates KDM5B, leading to decreased H4K20me3 and subsequent increase of GABPA expression, leading to reduced ROS and inhibition of ferroptosis. This finding reveals that ferroptosis regulation in LUAD is closely linked to ubiquitination imbalance and epigenetic dysregulation. Finally, we found increased cisplatin-induced ferroptosis when combined with GABPA suppression in KRAS-mutant LUAD organoids. These findings provide new insights for overcoming the challenge of platinum-based chemotherapy resistance in KRAS-mutant LUAD [3, 22].

## Materials and methods

### Cell lines and cell culture

Human KRAS-mutant LUAD cell lines A549 and SW1573 with KRAS driver mutations were obtained from the Shanghai Institute of Cell Biology, Chinese Academy of Sciences. All cell lines were cultured in RPMI-1640 medium (SB-C003, Share-bio, Shanghai) supplemented with 10% heat-inactivated fetal bovine serum (Ozfan, FBSKM0504), and the culture dishes (abs7005, absin) were placed in a humidified incubator at 37 °C with 5% CO₂. All cell lines were authenticated and routinely tested for mycoplasma contamination using a mycoplasma detection kit (K0103, HUABIO).

### Patient specimen

Human KRAS-mutant LUAD tissues were collected from the sample library of the Affiliated Cancer Hospital of Nanjing Medical University. The samples were frozen and stored in liquid nitrogen. The diagnosis of KRAS-mutant LUAD was confirmed based on clinical manifestation and pathological examination. Informed consent was obtained from all subjects.

### Cell counting Kit-8 cell proliferation assay

Cells were evenly seeded into 96-well plates (abs7243, absin), and different concentrations of drugs were added to the respective wells according to the experimental design, with at least three replicates per group. After incubation for 24 or 48 h, 10 μl of Cell Counting Kit-8 (CCK-8) reagent (SB-CCK8, Share-bio, Shanghai) was added to each well, followed by incubation in a dark environment at 37 °C for 1 h. Finally, each well’s absorbance (OD value) was measured at 450 nm using a microplate reader.

### Cytotoxicity assay

The cytotoxicity under different treatments was assessed using a lactate dehydrogenase (LDH) release kit (Beijing Boxbio Science & Technology Co., Ltd., China). The studies were carried out according to the instructions provided by the supplier.

### 2.5 5-ethynyl-2′-deoxyuridine assay

Cells were seeded into culture dishes and, upon reaching appropriate confluency, 5-ethynyl-2′-deoxyuridine (EdU) solution was added and incubated for an additional 4 h. Cells were washed with PBS, fixed, permeabilized, and incubated with an EdU fluorescent probe. Finally, cell proliferation was analyzed by flow cytometry.

### Western blot

After washing the collected cells with PBS, they were resuspended in RIPA lysis buffer containing protease inhibitors and lysed on ice for 30 min. The lysate was centrifuged at 12,000×*g* for 15 min at 4 °C, and the supernatant was collected to obtain the total protein sample. Protein concentration was determined using a BCA protein assay kit, and equal amounts of protein were loaded into each lane of a SurePAGETM precast gel (M00657, GenScript) after calculation. After separation by SDS-PAGE electrophoresis, proteins were transferred from the gel to a PVDF membrane. The membrane was then blocked with TBST containing 5% bovine serum albumin (BSA) at room temperature for 1 h. After blocking, the membrane was incubated with appropriately diluted primary antibody at 4 °C overnight. The next day, the membrane was washed three times with TBST for 10 min each, followed by incubation with the corresponding HRP-conjugated secondary antibody at room temperature for 1 h. After thorough washing again, the membrane was covered with ECL chemiluminescent substrate, and signals were captured using a chemiluminescence imaging system.

The protein extraction kit (EX1100) was purchased from Beijing Solarbio Science & Technology Co., Ltd. MOPS Running Buffer (SLB0091) was purchased from Smart-lifesciences, ChangZhou, China. SDS-PAGE Sample Loading Buffer (SB-PR037F), WB transfer buffer (SB-PR010), and BAS (SB-PRd069) were purchased from Share-bio, Shanghai, China. Anti-CASP1 (342947), anti-MLKL (R380559), anti-Bcl2 (R23309), anti-BAX (R380709) antibodies were purchased from Zen-Bioscience, China. Anti-RIPK3 (bsm-51714m) antibody was purchased from Bioss, USA. Anti-H3K9me3 (YP-Ab-01122), anti-H3K36me3 (YP-Ab-00812), anti-H3K27AC (YP-Ab-17840), anti-H3K4me3 (YP-Ab-01120) and anti-H3K79me3 (YP-Ab-00810) antibodies were purchased from UpingBio Technology Co., Ltd.; HangZhou, China. Anti-GAPDH (LF205) and anti-GPX4 (R011716) antibodies were acquired from Epizyme, Shanghai, China. Anti-SLC7A11, anti-FTH-1, anti-NRF2 (29650), anti-H3 (4499), and anti-H4 (13919) antibodies were acquired from Cell Signaling Technology, USA. Anti-GABPA (PA5-88319) and anti-OTUD7A (PA5-90565) antibodies were purchased from Thermo, USA. HRP-Goat Anti-Mouse (ZYID001-0050) and HRP-Goat Anti-Rabbit (ZYID002-0050) were purchased from ZUNYAN, Nanjing, China. Target proteins were visualized using the EZ ECL pico luminescence regent (AP34L025, Life-iLab, China).

### Real time-quantitative polymerase chain reaction

Total cellular RNA was extracted using an RNA extraction kit, and its concentration and purity were measured. Then, 1 μg of total RNA was taken to synthesize the first strand of cDNA using a reverse transcription kit. The cDNA template, specific primers, and SYBR Green qPCR master mix were combined, and nuclease-free water was added to a final volume of 20 μL. The program was run on a real-time quantitative PCR instrument, and a melting curve was finally plotted to verify amplification specificity. Using GAPDH as the internal reference, the relative expression of the target gene was calculated using the 2^(-ΔΔCt) method.

### Half-life experiment

Cells were inoculated into a 12-well plate, and once adhered, gradient plasmid transfection was performed. After 24 h of transfection, cells were treated with cycloheximide. Cells were harvested at various time intervals for subsequent WB analysis.

### In vivo ubiquitination experiment

Cells were transfected with His-KDM5B and Flag-OTUD7A plasmids, and treated with MG132 for 8 h before harvesting. Subsequently, after cell lysis, sonication, and centrifugation, one-tenth of the supernatant was taken to detect intracellular protein expression. The remaining supernatant was incubated with 1 μg of specific antibody under rotation at 4 °C for 3 h, followed by the addition of 40 μl Protein A/G magnetic beads and continued rotation at 4 °C for 8 h. The samples were placed on a magnetic stand to adsorb the beads, and the supernatant was discarded. The precipitate was washed three times with pre-cooled wash buffer, and finally resuspended in SDS-PAGE loading buffer and denatured by heating in a boiling water bath for subsequent WB analysis.

### Immunofluorescence experiment

Cells were seeded in plates containing cover slips. After full adhesion, they were gently washed twice with PBS, fixed with 4% paraformaldehyde at room temperature for 15 min, permeabilized with 0.2% Triton X-100 for 10 min, and blocked with 10% goat serum at room temperature for 1 h. After discarding the blocking solution, the diluted primary antibody was added directly and incubated at 4 °C overnight. The next day, cells were washed three times with PBST for 5 min each, then incubated with species-appropriate fluorescently labeled secondary antibody (SB-AB0142, SB-AB0151, Share-bio, Shanghai) at room temperature protected from light for 1 h. After thorough washing with PBST, nuclei were counterstained with diluted DAPI (SB-D4080, Share-bio, Shanghai) solution for 1 min at room temperature, protected from light. The cover slips were carefully removed, excess liquid was absorbed, and they were mounted upside down on glass slides containing anti-fade mounting medium. Finally, images were observed and captured using a laser scanning confocal microscope.

### BODIPY C11 staining

Cells were placed in the culture medium and treated with a 10 μM BODIPY C11working solution (diluted in HBSS). After incubation at 37 °C for 30 min, the cells were washed three times with PBS. Finally, detection was performed using a flow cytometer.

### JC-1 staining

Cells were collected by trypsinization, PBS washing, and centrifugation, and then resuspended in a working solution consisting of culture medium mixed with JC-1 (Beijing Solarbio Science & Technology Co., Ltd., M8650) staining solution at a 1:1 ratio. After incubation at 37 °C for 20 min, the cells were washed three times with ice-cold staining buffer. Finally, detection was performed using a flow cytometer.

### MitoSOX staining

After collecting the cells, the cell pellet was washed once with HBSS balanced salt solution. Subsequently, cells were resuspended using a 5% MitoSox diluted with HBSS. After incubating at 37 °C protected from light for 10 min, the cell pellet was washed three times with pre-warmed HBSS. Finally, detection was carried out using a flow cytometer.

### Construction of KDM5B, OTUD7A, and GABPA knockdown and overexpression stable cell lines in LUAD

A549 and SW1573 cells were transfected with lentiviruses (Shanghai Morzan Biotechnology Co., Ltd, MN-LTPK001, MN-LTPK003) for shRNA knockdown or overexpression of indicated genes. After transduction, cells were selected in medium containing 2 μg/mL puromycin to establish stable cell lines. WB analysis was subsequently performed to confirm knockdown or overexpression efficiency.

### ChIP-Seq experiment

ChIP Kit (26157) was purchased from Thermo, USA. Healthy growing cells were cross-linked with 1% formaldehyde at room temperature for 10 min, followed by the addition of glycine to terminate the reaction. After discarding the medium, cells were washed twice with ice-cold PBS, scraped off, and collected by centrifugation at 4 °C. Cells were resuspended in cell lysis buffer containing protease inhibitors and incubated on ice for 15 min. The nuclear pellet was resuspended in 200 μL of 1× ChIP buffer containing protease inhibitors and incubated on ice for 10 min. Chromatin was then sonicated using an ultrasonicator (power set to 85%) with the following parameters: three cycles of 10-s sonication with 30-s intervals, fragmenting DNA to 200–500 bp. The supernatant was collected 1% of the lysate was set aside as an input control. The remaining sample was equally divided into two parts, to which specific antibody and IgG isotype control antibody were added, respectively, followed by rotational incubation at 4 °C overnight. The next day, pretreated Protein A/G magnetic beads were added and incubated at 4 °C for 2 h. Bead-antibody complexes were captured using a magnetic stand, and the beads were washed sequentially with low-salt immunoprecipitation wash buffer and high-salt immunoprecipitation wash buffer. In total, 200 mM NaCl and Proteinase K were added to both the beads and input samples, and reverse cross-linking was performed at 65 °C for 1.5 h. Finally, DNA was purified using DNA purification columns, stored at −80 °C, and submitted for sequencing analysis. Anti-KDM5B (15327) and anti-H4K20me3 (5737) antibodies were acquired from Cell Signaling Technology, USA.

The ChIP-seq data analysis pipeline is as follows: raw data quality control was performed using fastp (v0.23.1), sequence alignment was conducted with Bowtie2 (v2.4.5) against the reference genome GRCh38, peak calling was performed using MACS2 (v2.2.7.1, q-value < 0.05), and peak annotation along with genomic region analysis was completed using ChIPseeker.

### Dual-luciferase reporter assay

Inoculate the successfully constructed overexpression vector bacterial suspension into 5 mL LB medium and incubate overnight at 37 °C with shaking. Seed cells in 24-well plates, and when cell confluence reaches approximately 70%, perform transfection experiments, replacing the medium with fresh medium 2 h before transfection. Take two EP tubes, add 25 μL opti-MEM to each, add 0.5 μg reporter plasmid and 0.05 μg pRL-TK reference plasmid to one tube, and add 1.5 μL GMTrans transfection reagent to the other tube, mix, and let stand at room temperature for 15 min to form complexes, then add dropwise to cells and continue culturing for 48 h. Discard the medium, wash with PBS, add lysis buffer to lyse for 15 min, centrifuge, and collect the supernatant. Take 50 μL supernatant, sequentially add 50 μL LAR II to measure firefly luciferase activity (Firefly RLU), then add 50 μL Stop & Glo® reagent to measure Renilla luciferase activity (Renilla RLU), using the ratio of the two as the relative reporter gene activity.

### Quantification and statistical analysis

All experiments were independently performed at least three times. Data are presented as mean ± standard deviation (SD). Statistical analyses were conducted using GraphPad Prism (USA). Statistical significance was determined using two-way ANOVA, and a *P* value < 0.05 was considered statistically significant. The sample size in this study was determined based on the results of previous related studies and experimental feasibility. Additionally, we conducted a post hoc power analysis using the obtained experimental data; the results showed that for the detected main effect (tumor volume change), the statistical power of the current sample size was greater than 0.8, indicating that the analysis possesses sufficient sensitivity.

### Subcutaneous tumor implantation mode

Four-week-old female BALB/c nude mice with an initial body weight of ~15 g (Jiangsu Jifa Pharmaceutical Co., Ltd., Jiangsu) were selected and randomly divided into six experimental groups. Subcutaneous tumor models were established on both sides of the nude mice using A549 and SW1573 cell lines (Vector/OE-OTUD7A/OE-KDM5B/OE-GABPA/OE-OTUD7A + OE-KDM5B+si-GABPA/si-GABPA, n = 5 per group). After washing the prepared cells with PBS, resuspend them in a mixture of PBS and Matrigel (KGL5101-5, Keygen BioTECH) solution at a 2:1 ratio, which was subsequently injected subcutaneously into the skin of 5-week-old nude mice. Another group of nude mice was randomly divided into four groups (Vector/Cisplatin/si-GABPA/Cisplatin+si-GABPA, *n* = 5 per group), and received subcutaneous injections of human KRAS-mutant LUAD organoid tumors. Cisplatin was administered via tail vein injection, and GABPA in vivo small interfering RNA was injected intratumorally (5 mg/kg every 5 days for a total of four doses). The recommended dose of GABPA in vivo siRNA was given simultaneously with Cisplatin, while the controls received an equal volume of PBS. All nude mice were weighed, and tumor dimensions were measured every 5 days. Finally, mice were euthanized using carbon dioxide, and the subcutaneous tumors were excised for size measurement. The tumor volume was calculated using the following formula: V = (length × width²)/2.

## Results

### KDM5B demethylates the H4K20me3 site to upregulate GABPA expression, promoting the proliferation of KRAS-mutant LUAD cell lines

**Fig. 1:**
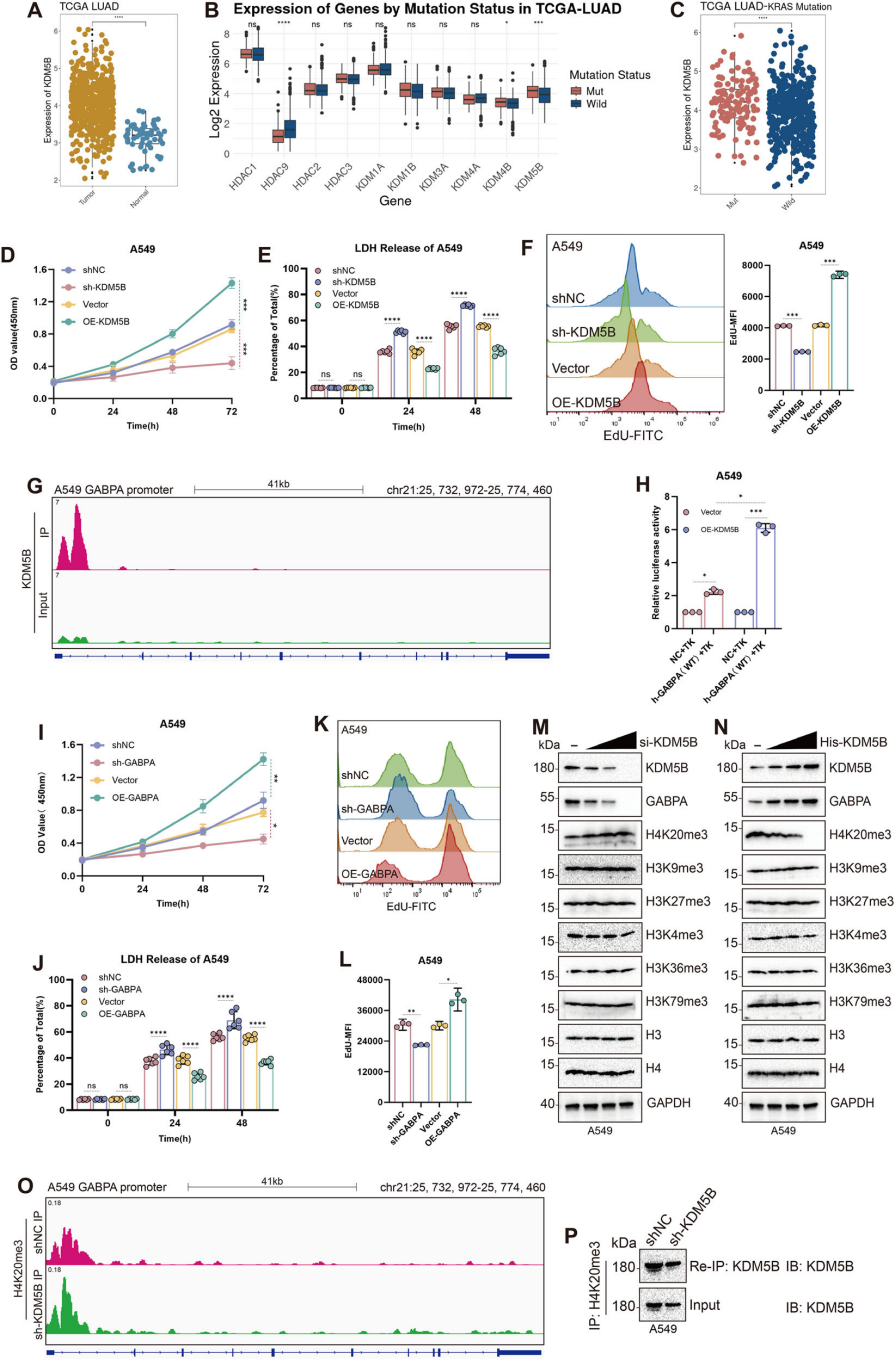
KDM5B demethylates the H4K20me3 site, upregulating GABPA expression and promoting the proliferation of KRAS-mutant LUAD cell lines. **A** Expression of KDM5B in TCGA-LUAD tumor and adjacent normal tissues. **B** Expression of various demethylases in KRAS-mutant versus wild-type LUAD. **C** Expression of KDM5B in KRAS-mutant versus wild-type LUAD. **D**, **E** The viability and mortality of cells were assessed at 24, 48, and 72 h following stable knockdown and overexpression of KDM5B in A549 cells using CCK-8 and LDH assays. **F** The proliferation rate of A549 cells was measured using the EdU assay following stable knockdown and overexpression of KDM5B. **G** ChIP-Seq was used to compare the whole-genome sequences of A549 cells with the DNA sequences retrieved through KDM5B protein binding pull-down assays. **H** In control and KDM5B-overexpressing A549 cells, GABPA expression plasmids were transfected, and changes in GABPA promoter transcriptional activity were detected using a dual-luciferase reporter system. **I**, **J** The viability and mortality of A549 cells were assessed at 24, 48, and 72 h following stable knockdown and overexpression of GABPA using CCK-8 and LDH assays. **K**, **L** The proliferation rate of A549 cells was measured using the EdU assay following stable knockdown and overexpression of GABPA. **M**, **N** In A549 cells, gradient transfection of KDM5B plasmids was conducted, and subsequent alterations in histone modifications were observed via WB. **O** In NC and KDM5B-knockdown A549 cells, ChIP-seq analysis was performed using an H4K20me3 antibody to compare the changes in H4K20me3 enrichment levels at the GABPA gene locus between the two groups. **P** WB was used to detect the KDM5B signal in H4K20me3 antibody IP and Input controls from NC and sh-KDM5B cells. All experimental procedures, except for ChIP-Seq, were independently repeated three times, showing consistent results. Error bars represent the mean ± S.D. *P < 0.05, **P < 0.01, ***P < 0.001, ****P < 0.0001.

In the TCGA-LUAD dataset, the expression of KDM5B was significantly higher in tumor tissues than in adjacent normal tissues. Furthermore, in KRAS-mutant LUAD, KDM5B expression was also substantially higher than in the KRAS wild-type group, and its basal expression level was higher than that of HDAC9, showing more pronounced statistical differences compared to KDM4B. Additionally, its expression is greater in KRAS-mutant LUAD samples compared to wild-type samples (Fig. 1A–C). Therefore, we constructed stable knockdown and overexpression cell lines of KDM5B in KRAS-mutant LUAD cell lines A549 and SW1573 (Fig. S1A, B). CCK-8 and EdU proliferation assay results indicated that overexpression of KDM5B significantly enhanced the cell proliferation, whereas KDM5B knockdown reduced the proliferation of both cell lines (Figs. 1D, F and S1C, E). The results of the LDH assay showed that the cell death rate in the sh-KDM5B group was significantly higher than that in the negative control (shNC) group, whereas the cell death rate in the OE-KDM5B group was significantly lower than that in the vector group (Figs. 1E and S1,D). To further explore the potential mechanism by which KDM5B promotes proliferation in KRAS-mutant LUAD cells, we conducted a KDM5B pulldown ChIP-Seq experiment in A549 cells and the expression of the top ten upregulated genes compared to the input in KDM5B knockdown and control cell lines, ultimately identifying GABPA as the most significantly regulated mRNA by KDM5B (Figs. 1G and S1,F). Using RT-PCR, we confirmed that overexpression of KDM5B enhanced the expression levels of GABPA (Fig. S1G). The dual-luciferase reporter assay results showed that KDM5B overexpression significantly enhanced the transcriptional activity of the GABPA promoter (Fig. 1H). Following this, we constructed stable knockdown and overexpression cell lines of GABPA in the A549 and SW1573 cell lines, and evaluated the efficiency of gene knockdown and overexpression by western blotting (WB) (Fig. S1,H). Subsequently, cell proliferation assays revealed that overexpression of GABPA significantly enhanced the proliferation capacity of KRAS-mutant LUAD cell lines, consistent with the oncogenic role of KDM5B (Figs. 1I–L and S1I–K).

According to previous reports, KDM5B primarily exerts its biological functions by removing methylation modifications from histone H3 [13, 23–25]. To precisely identify the specific demethylation sites of KDM5B in KRAS-mutant LUAD cell lines, we conducted WB detection of histone methylation at all common sites, revealing the most significant changes in H4K20me3. We conducted gradient transfection experiments in A549 cells, which showed that gradient overexpression of KDM5B led to a decrease in the expression gradient of histone H4K20me3, while gradient knockdown of KDM5B resulted in an increased expression gradient of H4K20me3 (Fig. 1M, N). We further compared the genome-wide distribution of H4K20me3 in shNC A549 cells and KDM5B-knockdown cells through ChIP-Seq using an H4K20me3 antibody. The results showed that after KDM5B knockdown, the H4K20me3 signal was significantly enhanced genome-wide and showed obvious enrichment in the GABPA promoter region (Fig. 1O). We performed WB analysis on the immunoprecipitated fractions from the ChIP assay. The results showed that KDM5B was specifically detected in the H4K20me3 antibody immunoprecipitated fraction, and this signal was significantly reduced in the sh-KDM5B group, confirming the functional association between KDM5B and H4K20me3 modification on chromatin (Fig. 1P). These findings indicated that KDM5B may elevate GABPA expression by demethylating H4K20me3.

### Inhibition of the KDM5B-GABPA axis promotes an increase in mitochondrial ROS in KRAS mutant LUAD cells and organoids, inducing ferroptosis and enhancing sensitivity to cisplatin

**Fig. 2:**
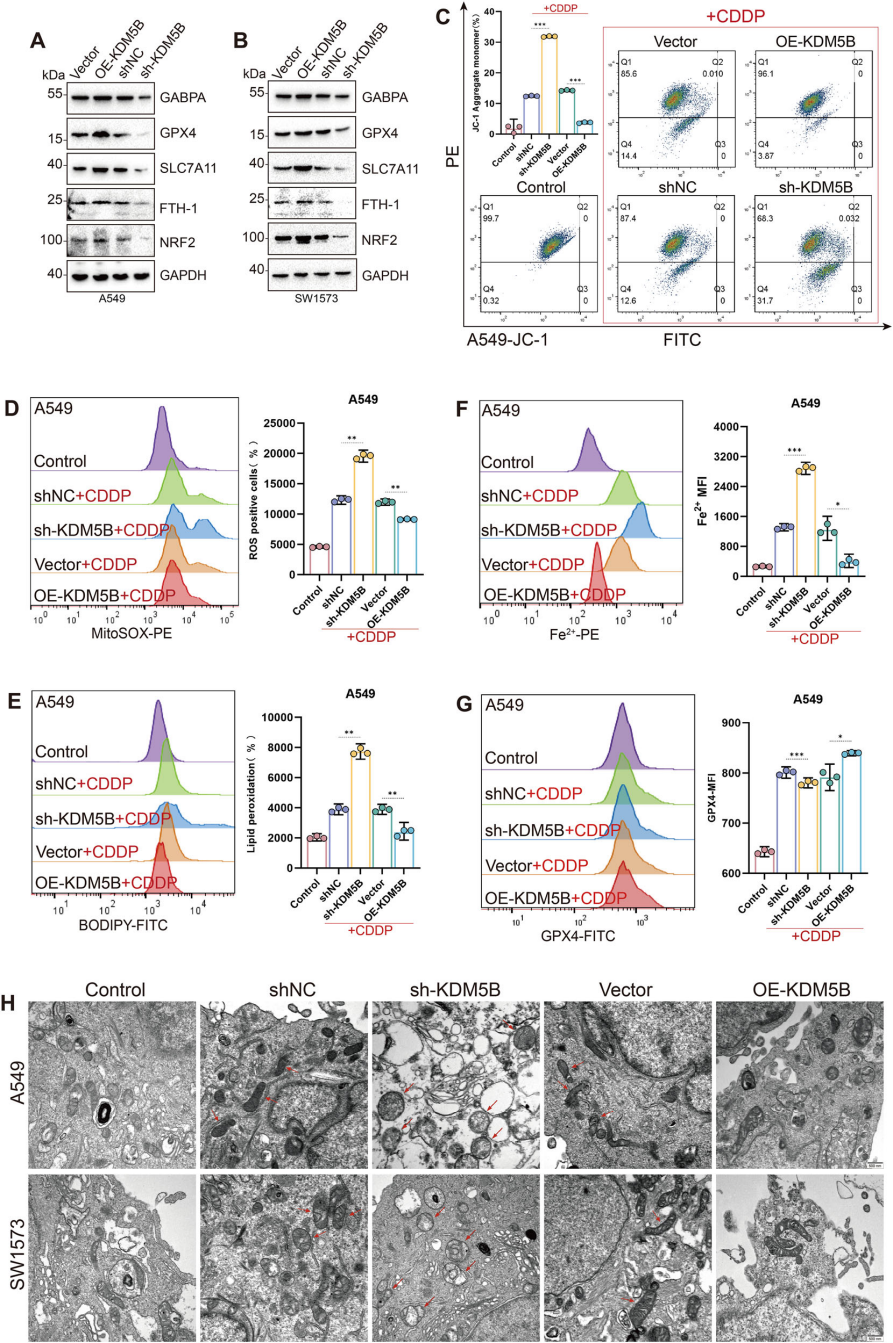
Inhibiting KDM5B promotes increased ROS production from mitochondria, thereby inducing ferroptosis and enhancing sensitivity to cisplatin. **A**, **B** Key markers of ferroptosis were detected in A549 and SW1573 LUAD cell lines using WB analysis. **C** The changes in the mitochondrial membrane potential of A549 cells are illustrated using the JC-1 probe after stable knockdown and overexpression of KDM5B. (All groups except the Control were pretreated with 20 μM cisplatin for 10 h, and **D**–**H** figures underwent identical treatment). **D**, **E** ROS and lipid ROS generation in A549 cells were detected using MitoSOX and BODIPY C11 probes after stable knockdown and overexpression of KDM5B. **F** In A549 cells, changes in intracellular Fe²⁺ levels were detected using an iron ion fluorescent probe. **G** Flow cytometry was employed to detect alterations in GPX4 expression levels in A549 cells following stable knockdown and overexpression of KDM5B. **H** Transmission electron microscopy imaging revealed alterations in mitochondrial morphology in A549 and SW1573 cells following KDM5B knockdown and overexpression. All experimental procedures were independently replicated three times, demonstrating consistent outcomes. Error bars represent the mean ± S.D. *P < 0.05, **P < 0.01, ***P < 0.001, ****P < 0.0001.

According to NCBI and related literature, GABPA plays an important regulatory role in cellular energy metabolism by activating cytochrome oxidase expression and regulating mitochondria-related nuclear genes [26]. The abnormal accumulation of ROS triggered by mitochondrial dysfunction exacerbates lipid peroxidation, constituting the core driving mechanism of ferroptosis. We speculate that GABPA may regulate cellular sensitivity to ferroptosis through the mitochondrial-oxidative stress axis [27]. To systematically investigate the regulatory role of the KDM5B-GABPA axis on cell death modalities, we analyzed the expression changes of key molecular markers of ferroptosis, apoptosis, necroptosis, and pyroptosis in A549 and SW1573 cells by WB. The results showed that among various cell death-related factors, the expression changes of key ferroptosis regulators SLC7A11, GPX4, FTH1, and NRF2 were the most significant, suggesting that KDM5B knockdown may inhibit the ferroptosis process in KRAS-mutant LUAD cells by downregulating GABPA (Figs. 2A, B and S1L, M).

**Fig. 3:**
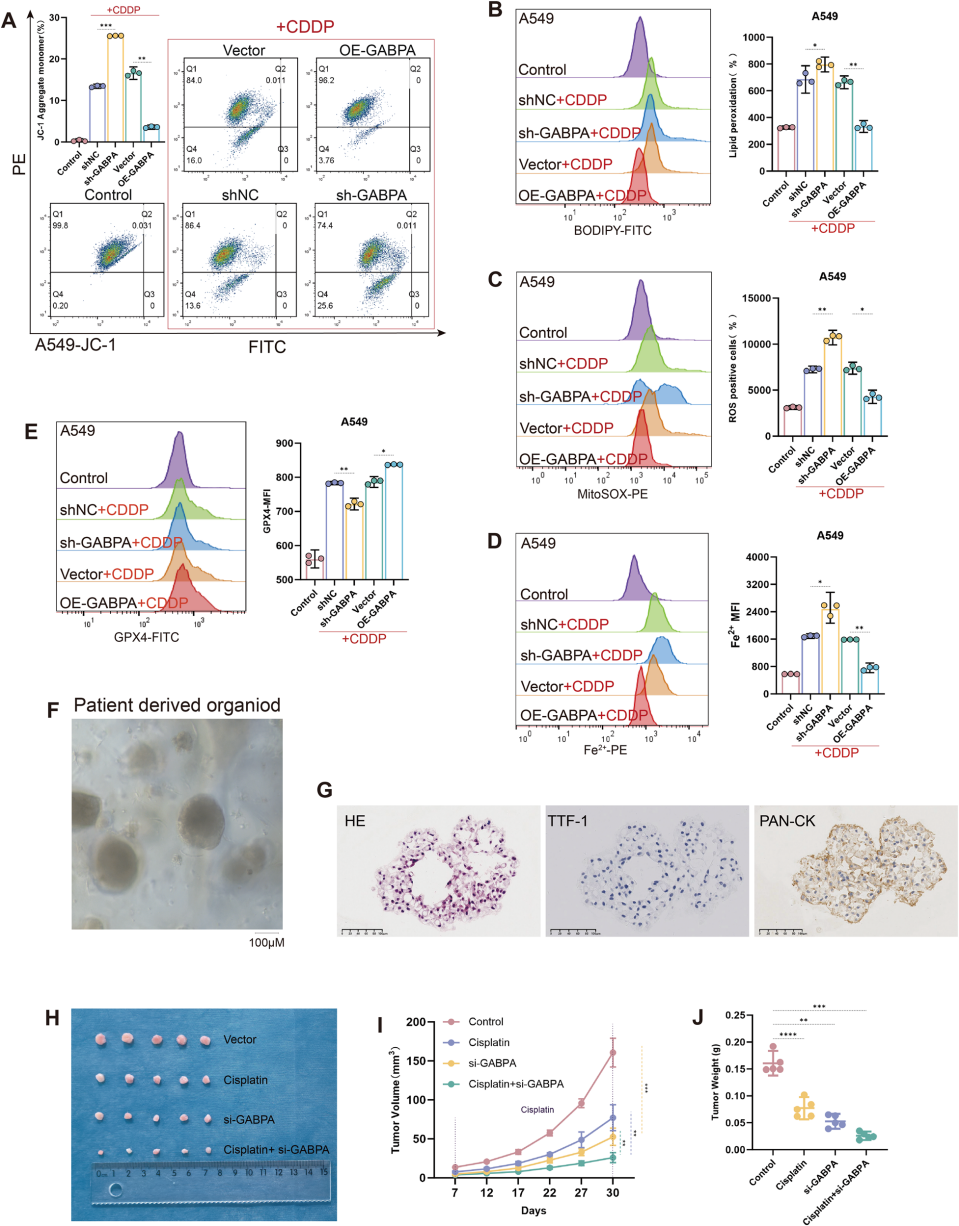
In KRAS-mutant LUAD cell lines and vivo organoid models, inhibiting GABPA promotes mitochondrial ROS production, thereby inducing ferroptosis and enhancing sensitivity to cisplatin. **A** The changes in the mitochondrial membrane potential of A549 cells are illustrated using the JC-1 probe after stable knockdown and overexpression of GABPA. (All groups except the Control were pretreated with 20 μM cisplatin for 10 h, and **B**–**E** underwent identical treatment). **B**, **C** The levels of ROS and lipid ROS generated within A549 cells were detected using BODIPY C11 and MitoSOX probes following stable knockdown and overexpression of GABPA. **D** In A549 cells, changes in intracellular Fe²⁺ levels were detected using an iron ion fluorescent probe. **E** Flow cytometry was employed to detect alterations in GPX4 expression levels in A549 cells following stable knockdown and overexpression of GABPA. **F**, **G** In vivo imaging of human lung-derived organoids, supplemented by HE and immunohistochemical staining for identification. **H**–**J** Growth assessments of xenograft tumors from human lung-derived organoids under normal conditions and after treatment with cisplatin and si-GABPA, with **I** illustrating the tumor volume changes and **J** providing a statistical overview of the final tumor weights. All experimental procedures were independently replicated three times, demonstrating consistent outcomes. Error bars represent the mean ± S.D. *P < 0.05, **P < 0.01, ***P < 0.001, ****P < 0.0001.

To further investigate the process and mechanism by which the KDM5B-GABPA axis regulates ferroptosis. We used the class II ferroptosis inducer cisplatin to induce ferroptosis in KRAS mutant LUAD cells [28–30]. Using flow cytometry with JC-1, BODIPY C11, divalent iron ion (Fe^2+^), and MitoSOX probes for analysis, we found that the knockdown of KDM5B and GABPA significantly decreased the mitochondrial membrane potential, along with a significant increase in intracellular ROS, Fe^2+^, and lipid peroxidation levels; in contrast, overexpression of KDM5B and GABPA significantly inhibited the generation of ROS, Fe^2+^, and lipid ROS, stabilized the mitochondrial membrane potential, and alleviated mitochondrial damage (Figs. 2C–F, 3A–D, S1N, O, and S2A–F). Further flow cytometry assessments revealed that the expression levels of GPX4 significantly decreased in the sh-KDM5B and sh-GABPA groups, whereas they significantly increased in the OE-KDM5B and OE-GABPA groups (Figs. 2G, 3E, and S2G, H). GPX4 is a key intracellular antioxidant enzyme that effectively inhibits ferroptosis by catalyzing the glutathione-dependent reduction of lipid peroxides. Transmission electron microscopy results showed that in the KDM5B knockdown group, the mitochondrial morphology was abnormal compared to the negative control group (shNC), with cristae membranes disappearing, suggesting possible ferroptosis [31–34] (Fig. 2H).

KRAS-mutant LUAD generates substantial ROS during tumorigenesis, which drives cancer cells to evolve a sophisticated and complex antioxidant response mechanism, resulting in their tolerance to targeted interventions against the antioxidant defense system [34–39]. This particular metabolic pathway and its reliance on antioxidant mechanisms render KRAS-mutant LUAD cells particularly vulnerable to interventions aimed at the ROS defense system, exhibiting selective cytotoxicity towards KRAS-mutant LUAD [40, 41]. We speculate that in KRAS-mutant LUAD, the combined application of cisplatin and ferroptosis inducers may disrupt the powerful redox balance system of tumor cells, leading to massive accumulation of ROS and lipid peroxides, which in turn causes severe mitochondrial damage and extensively induces tumor cell ferroptosis, ultimately enhancing the sensitivity of KRAS-mutant LUAD to cisplatin. To validate this hypothesis, we established a patient-derived KRAS-mutant LUAD organoid in vivo model based on nude mice. We identified that this model possesses LUAD pathological characteristics through HE staining and immunohistochemistry (Fig. 3F, G). Compared with cisplatin or si-GABPA alone, the combination of the two significantly improved the final tumor treatment outcome. The tumor volume and growth rate in the combination treatment group were significantly lower than those in the single-agent group (Figs. 3H–J and S3A, B).

### OTUD7A regulates ferroptosis in KRAS-mutant LUAD by interacting with KDM5B

**Fig. 4:**
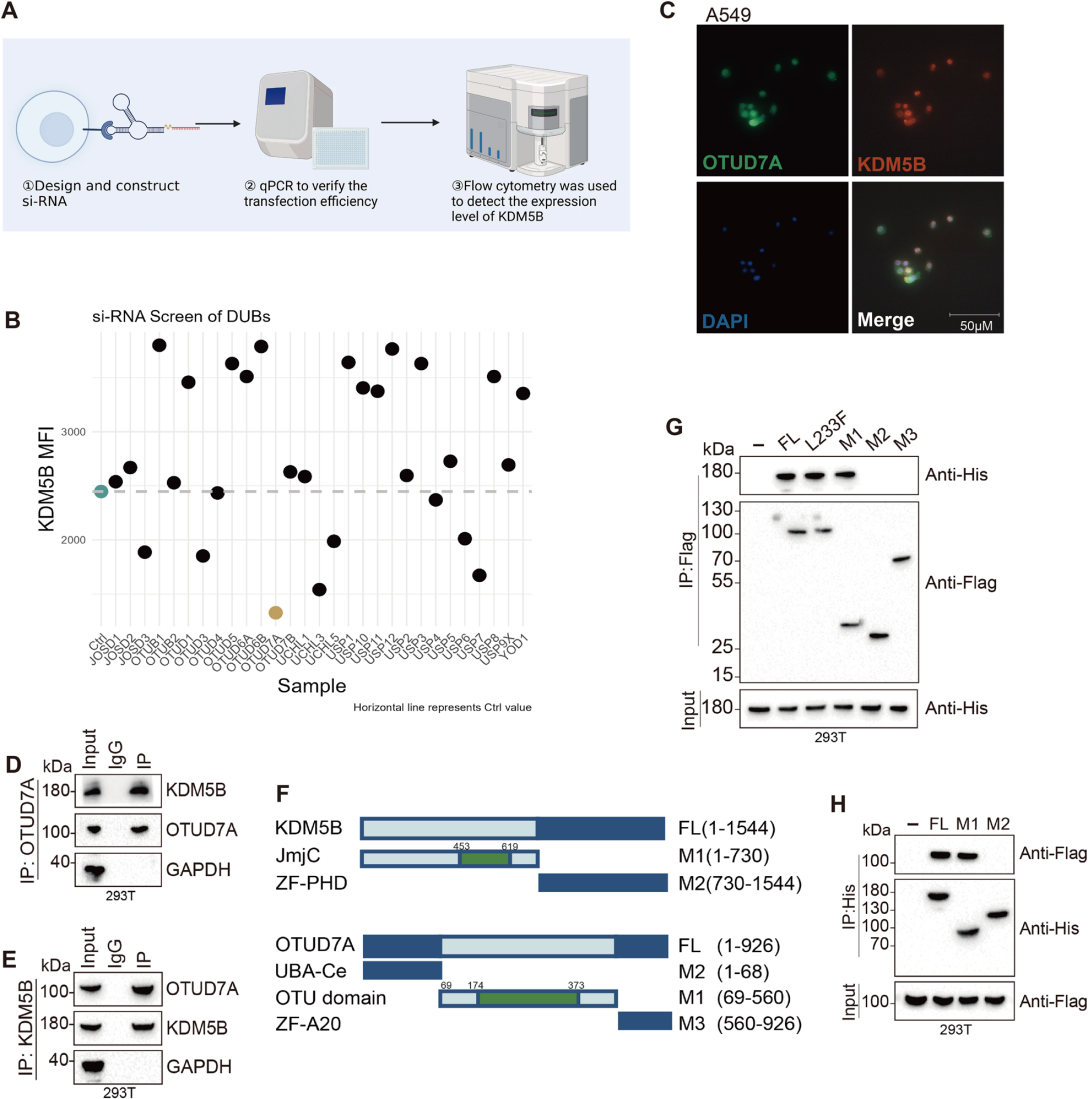
OTUD7A interacts with KDM5B. **A**, **B** Flow cytometric screening across the entire DUB family, with images displaying variations in KDM5B expression levels in A549 cells following DUB knockdown. **C** Immunofluorescence co-localization assays demonstrated co-localization of KDM5B and OTUD7A in A549 cells. **D**, **E** Co-immunoprecipitation experiments confirmed the interaction between KDM5B and OTUD7A in 293T cells. **F**–**H** Using the NCBI database, the domains of KDM5B and OTUD7A were identified, and corresponding truncated mutant plasmids were constructed; Co-IP experiments in 293T cells were performed to explore the interaction domains between these two molecules. All His-tags in this article are for the KDM5B protein, and Flag-tags are for OTUD7A. Error bars represent the mean ± S.D. *P < 0.05, **P < 0.01, ***P < 0.001, ****P < 0.0001.

We designed siRNAs targeting the entire DUB family for transient transfection in A549 cells to search for critical DUBs that regulate KDM5B. Following the validation of knockdown efficiency via RT-qPCR (Fig. S3C), we evaluated the expression levels of KDM5B after the knockdown of various deubiquitinases using flow cytometry. The results showed that the expression level of KDM5B was lowest in the transient knockout of OTU domain protein 7A (OTUD7A) (Fig. 4A, B). We then studied the mechanism by which OTUD7A regulates KDM5B, constructed stable knockdown and overexpression cell lines of OTUD7A in A549 and SW1573 cells, and confirmed the knockdown efficiency (Fig. S3I, J).

**Fig. 5:**
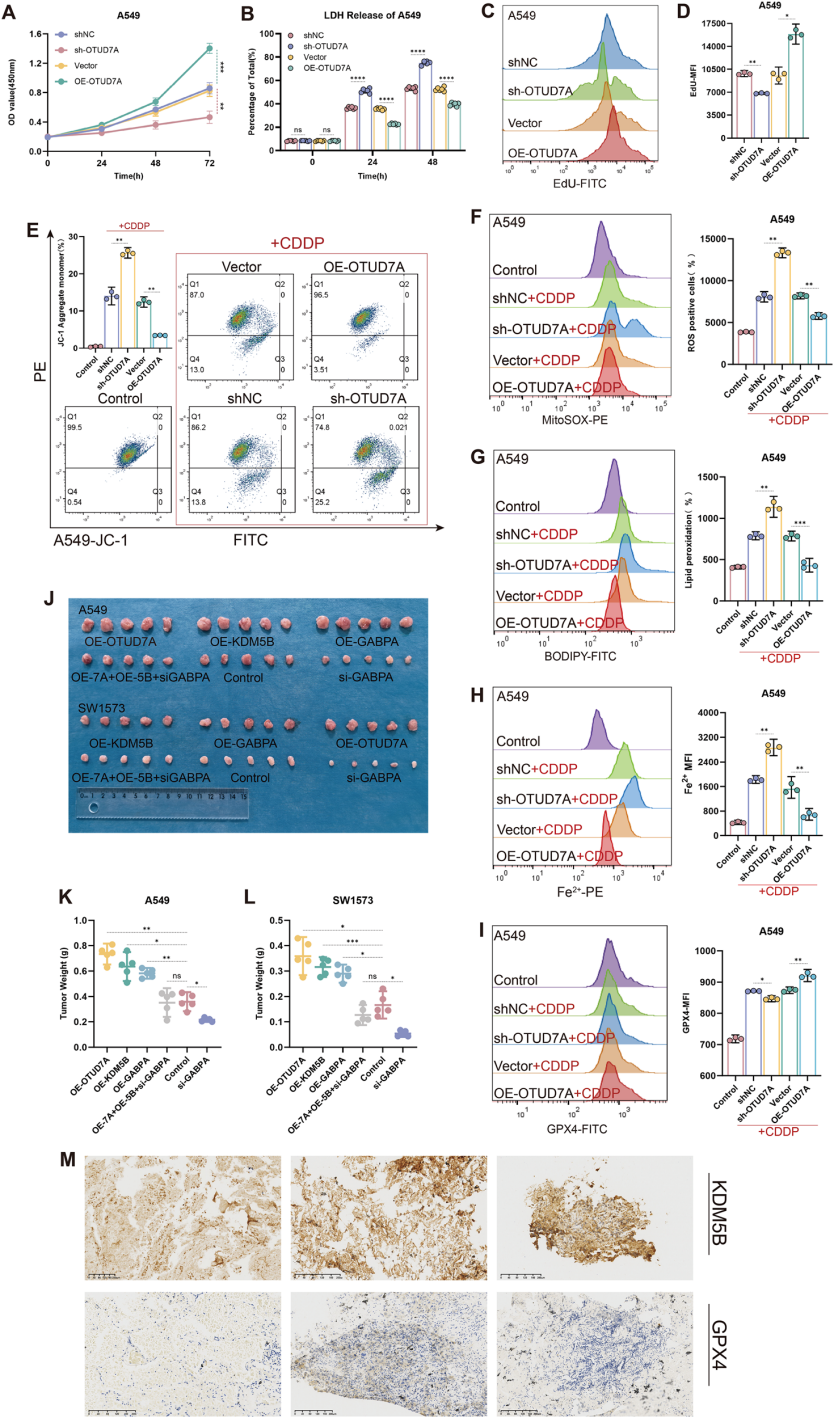
In KRAS mutant LUAD cell lines and mouse models, OTUD7A participates in the KDM5B-GABPA axis, affecting ferroptosis and sensitivity to cisplatin. **A**, **B** The viability and mortality of A549 cells were assessed at 24, 48, and 72 h following the stable knockdown and overexpression of OTUD7A using CCK-8 and LDH assays. **C**, **D** The proliferation rate of A549 cells was measured using the EdU assay following stable knockdown and overexpression of OTUD7A. **E** The changes in mitochondrial membrane potential of A549 cells were evaluated using the JC-1 probe following stable knockdown and overexpression of OTUD7A. (All groups except the Control were pretreated with 20 μM cisplatin for 10 h, and **F**–**I** figures underwent identical treatment). **F**, **G** The production of ROS and lipid ROS in A549 cells was detected using MitoSOX and BODIPY C11 probes after stable knockdown and overexpression of OTUD7A. **H** In A549 cells, changes in intracellular Fe²⁺ levels were detected using an iron ion fluorescent probe. **I** Flow cytometry was employed to investigate changes in GPX4 expression following stable knockdown and overexpression of OTUD7A in A549 cells. **J**–**L** A subcutaneous bilateral tumor model was established using A549 and SW1573 cell lines, with **K**, **L** presenting the final weight statistics of the tumors generated from A549 and SW1573 cells, respectively. **M** Immunohistochemical staining for KDM5B and GPX4 was performed on tissues from KRAS-mutant LUAD patients. All experimental procedures were independently replicated three times, demonstrating consistent outcomes. Error bars represent the mean ± S.D. *P < 0.05, **P < 0.01, ***P < 0.001, ****P < 0.0001.

**Fig. 6:**
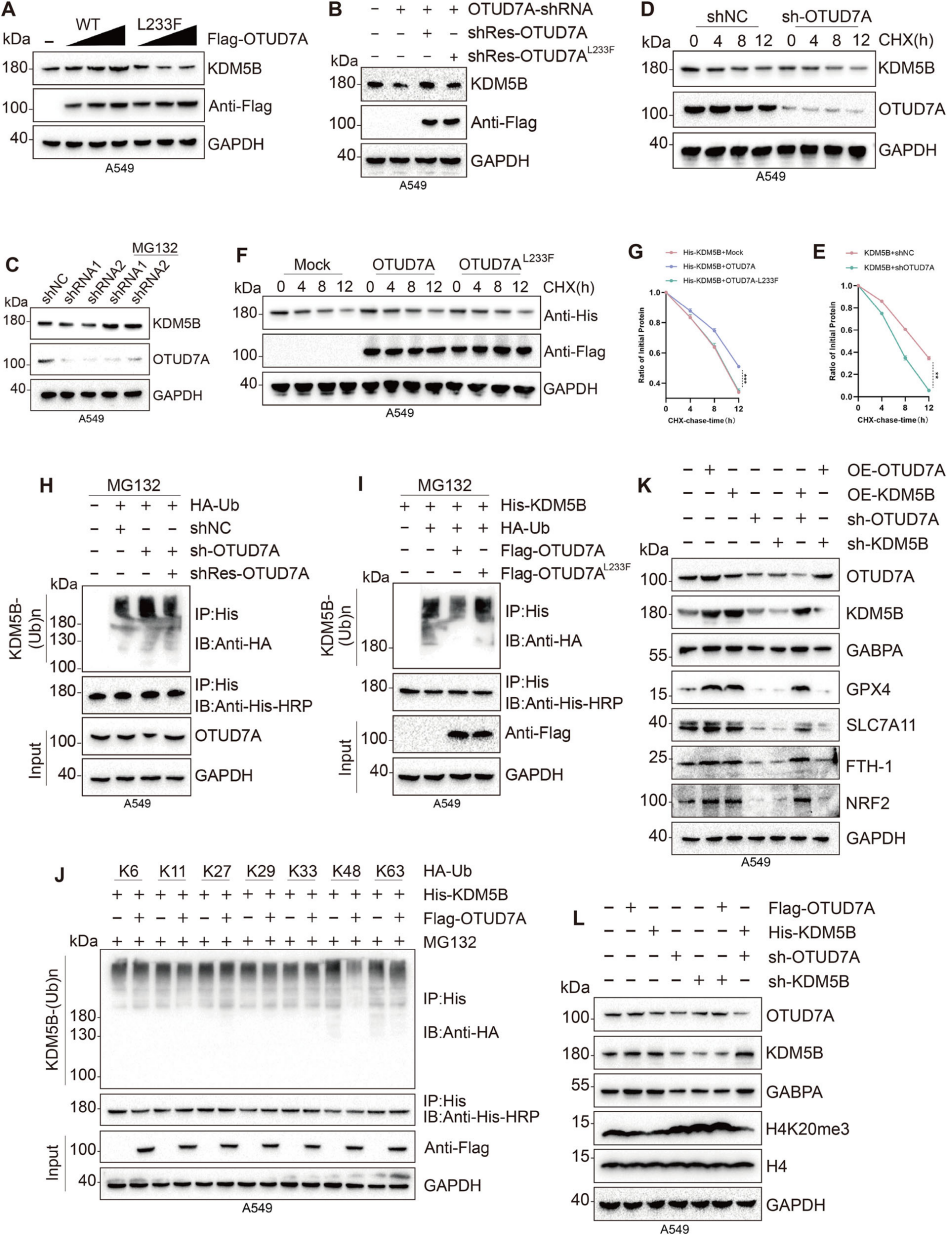
OTUD7A deubiquitinates KDM5B, stabilizing protein expression levels. **A** Following gradient transfection of normal and mutated OTUD7A in A549 cells, changes in KDM5B expression levels were analyzed via WB. **B** The impact on KDM5B expression levels in A549 cells was examined after OTUD7A knockdown alongside the transfection of wild-type or mutated OTUD7A plasmids, using WB analysis. **C** The variation in KDM5B expression levels was assessed after OTUD7A knockdown in A549 cells, with simultaneous administration of MG132. **D**–**G** In A549 cells, a half-life experiment was conducted, with WB used to verify how the expression levels of KDM5B change over time following the addition of actinomycin D, after OTUD7A knockdown or overexpression, and in cells overexpressing mutated OTUD7A. **H**, **I** In vivo ubiquitination experiments conducted in A549 cells investigated changes in KDM5B protein levels under conditions of OTUD7A overexpression, impaired OTUD7A overexpression, or OTUD7A knockdown with transfection of the wild-type OTUD7A plasmid. **J** In vivo ubiquitination experiments in A549 cells revealed the changes in ubiquitination sites during the interaction between OTUD7A and KDM5B. **K** The expression changes of GPX4, SLC7A11, FTH-1, and NRF2 resulting from the OTUD7A-KDM5B-GABPA axis were detected in A549 cells via WB. **L** The influence of the OTUD7A-KDM5B-GABPA axis on histone modification at H4K20me3 was assessed by WB analysis. Error bars represent the mean ± S.D. *P < 0.05, **P < 0.01, ***P < 0.001, ****P < 0.0001.

Co-IP experiments performed in SW1573, A549, and 293T cell lines, as well as immunofluorescence co-localization experiments in SW1573 and A549 cell lines, demonstrated a significant interaction between OTUD7A and KDM5B (Figs. 4C–E and S3D–H). To accurately elucidate the interaction mechanism and interaction domains between OTUD7A and KDM5B, we constructed truncated mutant plasmids of KDM5B and OTUD7A based on the conserved domain predictions from NCBI. The L233F mutation in OTUD7A is considered a loss-of-function variant [42]. The experimental results showed specific binding between the Jmjc domain of KDM5B and the OTU domain of OTUD7A (Fig. 4F–H). Using CCK-8, LDH, and EdU assays, we observed that the OTUD7A overexpression group showed significantly faster cell proliferation and a substantial decrease in the cell death rate compared to the negative control group (Vector) (Figs. 5A–D and S3K–M). Flow cytometry analysis of JC-1, BODIPY C11, Fe^2+^, and MitoSOX indicated that the overexpression of OTUD7A significantly reduced mitochondria-derived ROS, intracellular Fe²⁺ levels, and decreased the accumulation of lipid peroxidation products, and alleviated mitochondrial damage (Figs. 5E–H, S3O and S4A–C). Moreover, the expression level of GPX4 was elevated in response to OTUD7A overexpression (Figs. 5I and S3N). The above conclusions were further validated through WB experiments, which revealed that overexpression of OTUD7A enhanced the steady-state levels of KDM5B and strengthened its demethylation capability towards H4K20me3 (Figs. 6L and S5J), leading to the upregulation of GABPA expression and a subsequent significant increase in the expression of GPX4, SLC7A11, FTH-1, and NRF2, a process that is mediated by KDM5B (Figs. 6K and S5L). Consequently, we concluded that OTUD7A regulates the GABPA axis mediated by KDM5B, thereby inhibiting ferroptosis in KRAS-mutant LUAD cell lines.

Results from in vivo experiments indicated that the tumor proliferation rate in the groups overexpressing OTUD7A, KDM5B, and GABPA was significantly greater than that of the negative control group (Vector), and tumor volume was significantly increased. The tumor proliferation rate and volume in the OE-OTUD7A + OE-KDM5B + si-GABPA group showed no significant differences compared to the Vector group, suggesting that the effects of OTUD7A and KDM5B on KRAS-mutant LUAD are mediated by GABPA. Furthermore, the tumor proliferation rate in the si-GABPA group was significantly slowed, and tumor volume was markedly reduced (Figs. 5J–L and S4D–G). Meanwhile, we further analyzed the clinical samples. In KRAS-mutant LUAD patient tissues, immunohistochemical staining results confirmed the significantly high expression of KDM5B, accompanied by a synchronous increase in GPX4 expression levels.

### OTUD7A specifically removes ubiquitin from the K48 position, facilitating the deubiquitination of KDM5B, thereby stabilizing the expression level of the KDM5B protein

To further explore the mechanism by which OTUD7A stabilizes the KDM5B protein, we performed gradient transfection experiments, and based on reports by Uddin [43]. We gradient-transfected wild-type and L233F mutant OTUD7A and found that wild-type OTUD7A could stabilize the KDM5B protein, while the loss-of-function OTUD7A did not possess this function (Figs. 6A and S5A). Follow-up rescue experiments, specifically transfecting wild-type and mutant OTUD7A plasmids into OTUD7A knockdown cells, further validated the corresponding results (Figs. 6B and S5G). Half-life experiments showed that in the control group (shNC), the degradation of KDM5B protein increased over time, while in the sh-OTUD7A group, the degradation of KDM5B was exacerbated and the half-life was significantly shortened, indicating that OTUD7A can effectively stabilize the expression of KDM5B (Figs. 6D, E and S5B, C). Further comparison of wild-type and loss-of-function OTUD7A transfections revealed that KDM5B degradation in the non-functional OTUD7A group did not change. In contrast, degradation in the wild-type OTUD7A group was significantly reduced (Figs. 6F, G and S5, D, E). These results collectively indicate that OTUD7A maintains stable expression of KDM5B in KRAS-mutant LUAD cells, whereas non-functional OTUD7A lacks this ability.

OTUD7A has been identified as a deubiquitinase [44, 45]. We found that knocking down OTUD7A while simultaneously adding a proteasome inhibitor resulted in a decrease in KDM5B expression levels in the sh-OTUD7A group compared to the negative control group (shNC). Conversely, in the group where MG132 was added under the same conditions, KDM5B levels increased, which suggests that OTUD7A relies on the proteasome to exert its function and regulates KDM5B through the ubiquitin-proteasome pathway (Figs. 6C and S5F). Next, we performed an in vivo ubiquitination assay. In the in vivo ubiquitination assays using A549 and 293 T cells, the results indicated that the overexpression of OTUD7A significantly decreased the ubiquitination levels of KDM5B. In contrast, overexpression of the loss-of-function OTUD7A did not demonstrate significant changes. Additionally, the ubiquitination level of KDM5B in the sh-OTUD7A group was elevated, and transfection of the wild-type Rescue OTUD7A plasmid into this group restored KDM5B ubiquitination levels to normal (Figs. 6H, I and S5H, I). These results confirm that OTUD7A deubiquitinates KDM5B via the ubiquitin-proteasome pathway, thereby stabilizing its expression. Finally, to investigate the type of ubiquitin chain targeted by OTUD7A for KDM5B deubiquitination, we conducted in vivo ubiquitination assays. The results showed that K48-linked ubiquitin chains exhibited the most significant changes (Figs. 6J and S5K). In summary, our study demonstrates that OTUD7A specifically removes K48-linked ubiquitin chains from the KDM5B protein, thereby reducing its ubiquitination level and enhancing its protein stability.

## Discussion

This study reveals for the first time the critical role of the OTUD7A-KDM5B-GABPA signaling axis in regulating ferroptosis in KRAS-mutant lung adenocarcinoma. We found that the deubiquitinase OTUD7A stabilizes KDM5B by removing its K48-linked ubiquitin chains, thereby inhibiting proteasomal degradation. The stabilized KDM5B then binds to and demethylates the repressive histone mark H4K20me3 at the GABPA promoter, thereby relieving its transcriptional repression. This epigenetic reprogramming ultimately enhances the expression of GABPA target genes involved in mitochondrial function and antioxidant defense, suppressing ferroptosis and promoting tumor survival.

Our findings indicate that epigenetic regulatory mechanisms play a central role in ferroptosis within KRAS-driven lung adenocarcinoma. It is known that KRAS mutations can lead to patient resistance to conventional chemotherapeutic agents like cisplatin by remodeling cellular metabolic pathways and enhancing the antioxidant defense system [2, 34–37]. In contrast to previous studies that primarily focused on mechanisms of metabolic adaptation, this study is the first to uncover a novel epigenetic regulatory mechanism: the dynamic changes in H4K20me3 modification mediated by KDM5B are crucial for maintaining redox homeostasis. This discovery aligns with recent studies on the involvement of epigenetic regulators in ferroptosis [31, 46–50], but innovatively defines the OTUD7A-KDM5B axis as a direct bridge linking the regulation of protein stability to histone modification, thereby precisely controlling the ferroptosis process. It is noteworthy that ferroptosis inducers can synergize with cisplatin to produce a combined cytotoxic effect by promoting the massive generation of mitochondrial ROS [34, 37]. This discovery emphasizes the complexity of tumor cell survival mechanisms, and reveals a therapeutic vulnerability in KRAS-mutant tumor cells under specific targeted interventions, indicating that clinical approaches should incorporate a multidisciplinary strategy for more pronounced tumor regression effects [22, 51–54]. Our study elucidates the molecular mechanism underlying this synergy: disrupting the OTUD7A-KDM5B-GABPA signaling axis effectively dismantles the unique antioxidant defense system of KRAS-mutant cells, significantly weakening their antioxidant capacity and consequently enhancing their susceptibility to oxidative stress-induced death. Additionally, the OTUD7A-KDM5B-GABPA axis further emphasizes the interactions between epigenetics and protein stability within the tumor microenvironment [55–57]. Future research on tumors with similar genetic backgrounds should investigate the generalizability of this mechanism in greater detail.

While this research has clearly defined the regulatory relationship among OTUD7A, KDM5B, and GABPA in cell and animal models, additional clinical samples and prolonged observations are required to evaluate the roles and regulatory mechanisms of KDM5B and its downstream molecules in the ferroptosis process of KRAS-mutant LUAD. Conversely, although we investigated the influence of OTUD7A on KDM5B stability and GABPA expression, it merits further exploration whether the functions of KDM5B, GABPA, and OTUD7A vary under different microenvironmental conditions (such as hypoxia and inflammation) [58–61], as well as the potential presence of other feedback mechanisms or regulatory factors.

Finally, we revealed the mechanism by which KRAS-mutant LUAD develops resistance to ferroptosis due to epigenetic dysregulation. At the same time, we investigated the therapeutic strategy of co-administering cisplatin with ferroptosis inducers through human organoid tumor models, providing mechanistic support for the combined application of ferroptosis induction and cisplatin in treating KRAS-mutant LUAD. This work not only deepens our understanding of the intricate connections between protein stability, epigenetics, and metabolic cell death but also paves the way for novel combination therapies designed to overcome treatment resistance in this challenging subset of lung cancer.

## Supplementary information

supplementary material

original data

## Data Availability

The sequencing datasets supporting the conclusions of this article are available in the NCBI Sequence Read Archive (SRA) repository, under the BioProject accession [PRJNA1353613] and [PRJNA1353647].

## References

[1] Bray F, Laversanne M, Sung H, Ferlay J, Siegel RL, Soerjomataram I, et al. Global cancer statistics 2022: GLOBOCAN estimates of incidence and mortality worldwide for 36 cancers in 185 countries. CA Cancer J Clin. 2024;74:229–63. 10.3322/caac.21834. 38572751 10.3322/caac.21834

[2] Punekar SR, Velcheti V, Neel BG, Wong K-K. The current state of the art and future trends in RAS-targeted cancer therapies. Nat Rev Clin Oncol. 2022;19:637–55. 10.1038/s41571-022-00671-9. 36028717 10.1038/s41571-022-00671-9 PMC9412785

[3] Canon J, Rex K, Saiki AY, Mohr C, Cooke K, Bagal D, et al. The clinical KRAS(G12C) inhibitor AMG 510 drives anti-tumour immunity. Nature. 2019;575:217–23. 10.1038/s41586-019-1694-1. 31666701 10.1038/s41586-019-1694-1

[4] Herbst RS, Morgensztern D, Boshoff C. The biology and management of non-small cell lung cancer. Nature. 2018;553:446–54. 10.1038/nature25183. 29364287 10.1038/nature25183

[5] Hendriks LE, Kerr KM, Menis J, Mok TS, Nestle U, Passaro A, et al. Oncogene-addicted metastatic non-small-cell lung cancer: ESMO Clinical Practice Guideline for diagnosis, treatment and follow-up✰. Ann Oncol. 2023;34:339–57. 10.1016/j.annonc.2022.12.009. 36872130 10.1016/j.annonc.2022.12.009

[6] Prior IA, Hood FE, Hartley JL. The frequency of Ras mutations in cancer. Cancer Res. 2020;80:2969–74. 10.1158/0008-5472.CAN-19-3682. 32209560 10.1158/0008-5472.CAN-19-3682 PMC7367715

[7] Dimitrova E, Turberfield AH, Klose RJ. Histone demethylases in chromatin biology and beyond. EMBO Rep. 2015;16:1620–39. 10.15252/embr.201541113. 26564907 10.15252/embr.201541113 PMC4687429

[8] Gunn K, Myllykoski M, Cao JZ, Ahmed M, Huang B, Rouaisnel B. et al. (R)-2-Hydroxyglutarate Inhibits KDM5 Histone Lysine Demethylases to Drive Transformation in IDH-Mutant Cancers. Cancer Discov. 2023;13:1478–1497. 10.1158/2159-8290.CD-22-0825. 36847506 10.1158/2159-8290.CD-22-0825 PMC10238656

[9] Verma A, Singh A, Singh MP, Nengroo MA, Saini KK, Satrusal SR, et al. EZH2-H3K27me3 mediated KRT14 upregulation promotes TNBC peritoneal metastasis. Nat Commun. 2022;13:7344. 10.1038/s41467-022-35059-x. 36446780 10.1038/s41467-022-35059-x PMC9708848

[10] Marsolier J, Prompsy P, Durand A, Lyne A-M, Landragin C, Trouchet A, et al. H3K27me3 conditions chemotolerance in triple-negative breast cancer. Nat Genet. 2022;54:459–68. 10.1038/s41588-022-01047-6. 35410383 10.1038/s41588-022-01047-6 PMC7612638

[11] Gou, D, Liu, R, Shan, X, Deng, H, Chen, C, Xiang, J, et al. Gluconeogenic enzyme PCK1 supports S-adenosylmethionine biosynthesis and promotes H3K9me3 modification to suppress hepatocellular carcinoma progression. J Clin Invest 2023. 10.1172/JCI161713. 10.1172/JCI161713 PMC10313362 37166978

[12] Zhang S-M, Cai WL, Liu X, Thakral D, Luo J, Chan LH, et al. KDM5B promotes immune evasion by recruiting SETDB1 to silence retroelements. Nature. 2021;598:682–7. 10.1038/s41586-021-03994-2. 34671158 10.1038/s41586-021-03994-2 PMC8555464

[13] Xhabija B, Kidder BL. KDM5B is a master regulator of the H3K4-methylome in stem cells, development and cancer. Semin Cancer Biol. 2019;57:79–85. 10.1016/j.semcancer.2018.11.001. 30448242 10.1016/j.semcancer.2018.11.001 PMC6522339

[14] Zhang B, Li J, Wang Y, Liu X, Yang X, Liao Z, et al. Deubiquitinase USP7 stabilizes KDM5B and promotes tumor progression and cisplatin resistance in nasopharyngeal carcinoma through the ZBTB16/TOP2A axis. Cell Death Differ. 2024;31:309–21. 10.1038/s41418-024-01257-x. 38287116 10.1038/s41418-024-01257-x PMC10923876

[15] Zhang Y, Gao Y, Jiang Y, Ding Y, Chen H, Xiang Y, et al. Histone demethylase KDM5B licenses macrophage-mediated inflammatory responses by repressing Nfkbia transcription. Cell Death Differ. 2023;30:1279–92. 10.1038/s41418-023-01136-x. 36914768 10.1038/s41418-023-01136-x PMC10154333

[16] Xiang Y, Zhu Z, Han G, Ye X, Xu B, Peng Z, et al. JARID1B is a histone H3 lysine 4 demethylase up-regulated in prostate cancer. Proc Natl Acad Sci USA. 2007;104:19226–31. 10.1073/pnas.0700735104. 18048344 10.1073/pnas.0700735104 PMC2148272

[17] Hayami S, Yoshimatsu M, Veerakumarasivam A, Unoki M, Iwai Y, Tsunoda T, et al. Overexpression of the JmjC histone demethylase KDM5B in human carcinogenesis: involvement in the proliferation of cancer cells through the E2F/RB pathway. Mol Cancer. 2010;9:59. 10.1186/1476-4598-9-59. 20226085 10.1186/1476-4598-9-59 PMC2848192

[18] Liu F, Chen J, Li K, Li H, Zhu Y, Zhai Y, et al. Ubiquitination and deubiquitination in cancer: from mechanisms to novel therapeutic approaches. Mol Cancer. 2024;23:148. 10.1186/s12943-024-02046-3. 39048965 10.1186/s12943-024-02046-3 PMC11270804

[19] Cockram PE, Kist M, Prakash S, Chen S-H, Wertz IE, Vucic D. Ubiquitination in the regulation of inflammatory cell death and cancer. Cell Death Differ. 2021;28:591–605. 10.1038/s41418-020-00708-5. 33432113 10.1038/s41418-020-00708-5 PMC7798376

[20] Popovic D, Vucic D, Dikic I. Ubiquitination in disease pathogenesis and treatment. Nat Med. 2014;20:1242–53. 10.1038/nm.3739. 25375928 10.1038/nm.3739

[21] Dewson G, Eichhorn PJA, Komander D. Deubiquitinases in cancer. Nat Rev Cancer. 2023;23:842–62. 10.1038/s41568-023-00633-y. 37935888 10.1038/s41568-023-00633-y

[22] Hanahan D. Hallmarks of cancer: new dimensions. Cancer Discov. 2022;12:31–46. 10.1158/2159-8290.CD-21-1059. 35022204 10.1158/2159-8290.CD-21-1059

[23] He R, Xhabija B, Gopi LK, Kurup JT, Xu Z, Liu Z, et al. H3K4 demethylase KDM5B regulates cancer cell identity and epigenetic plasticity. Oncogene. 2022;41:2958–72. 10.1038/s41388-022-02311-z. 35440714 10.1038/s41388-022-02311-z PMC9426628

[24] Xu W, Zhou B, Zhao X, Zhu L, Xu J, Jiang Z, et al. KDM5B demethylates H3K4 to recruit XRCC1 and promote chemoresistance. Int J Biol Sci. 2018;14:1122–32. 10.7150/ijbs.25881. 29989047 10.7150/ijbs.25881 PMC6036731

[25] Kidder BL, Hu G, Zhao K. KDM5B focuses H3K4 methylation near promoters and enhancers during embryonic stem cell self-renewal and differentiation. Genome Biol. 2014;15:R32. 10.1186/gb-2014-15-2-r32. 24495580 10.1186/gb-2014-15-2-r32 PMC4053761

[26] Kang YK, Putluri N, Maity S, Tsimelzon A, Ilkayeva O, Mo Q, et al. CAPER is vital for energy and redox homeostasis by integrating glucose-induced mitochondrial functions via ERR-α-Gabpa and stress-induced adaptive responses via NF-κB-cMYC. PLoS Genet. 2015;11:e1005116 10.1371/journal.pgen.1005116. 25830341 10.1371/journal.pgen.1005116 PMC4382186

[27] George M, Reddy AP, Reddy PH, Kshirsagar S. Unraveling the NRF2 confusion: distinguishing nuclear respiratory factor 2 from nuclear erythroid factor 2. Ageing Res Rev. 2024;98:102353. 10.1016/j.arr.2024.102353. 38815934 10.1016/j.arr.2024.102353 PMC11176867

[28] Liu Z, Zhang H, Hong G, Bi X, Hu J, Zhang T, et al. Inhibition of Gpx4-mediated ferroptosis alleviates cisplatin-induced hearing loss in C57BL/6 mice. Mol Ther. 2024;32:1387–406. 10.1016/j.ymthe.2024.02.029. 38414247 10.1016/j.ymthe.2024.02.029 PMC11081921

[29] Guo J, Xu B, Han Q, Zhou H, Xia Y, Gong C, et al. Ferroptosis: a novel anti-tumor action for cisplatin. Cancer Res Treat. 2018;50:445–60. 10.4143/crt.2016.572. 28494534 10.4143/crt.2016.572 PMC5912137

[30] Wang L, Chen X, Yan C. Ferroptosis: an emerging therapeutic opportunity for cancer. Genes Dis. 2022;9:334–46. 10.1016/j.gendis.2020.09.005. 35224150 10.1016/j.gendis.2020.09.005 PMC8843872

[31] Qiu S, Zhong X, Meng X, Li S, Qian X, Lu H, et al. Mitochondria-localized cGAS suppresses ferroptosis to promote cancer progression. Cell Res. 2023;33:299–311. 10.1038/s41422-023-00788-1. 36864172 10.1038/s41422-023-00788-1 PMC10066369

[32] Bi Y, Liu S, Qin X, Abudureyimu M, Wang L, Zou R, et al. FUNDC1 interacts with GPx4 to govern hepatic ferroptosis and fibrotic injury in a mitophagy-dependent manner. J Adv Res. 2024;55:45–60. 10.1016/j.jare.2023.02.012. 36828120 10.1016/j.jare.2023.02.012 PMC10770120

[33] Tadokoro T, Ikeda M, Ide T, Deguchi H, Ikeda S, Okabe K, et al. Mitochondria-dependent ferroptosis plays a pivotal role in doxorubicin cardiotoxicity. JCI Insight. 2020;5:e132747–132747. 10.1172/jci.insight.132747. 32376803 10.1172/jci.insight.132747 PMC7253028

[34] Weinberg F, Hamanaka R, Wheaton WW, Weinberg S, Joseph J, Lopez M, et al. Mitochondrial metabolism and ROS generation are essential for Kras-mediated tumorigenicity. Proc Natl Acad Sci USA. 2010;107:8788–93. 10.1073/pnas.1003428107. 20421486 10.1073/pnas.1003428107 PMC2889315

[35] Kerr EM, Gaude E, Turrell FK, Frezza C, Martins CP. Mutant Kras copy number defines metabolic reprogramming and therapeutic susceptibilities. Nature. 2016;531:110–3. 10.1038/nature16967. 26909577 10.1038/nature16967 PMC4780242

[36] Yun J, Rago C, Cheong I, Pagliarini R, Angenendt P, Rajagopalan H, et al. Glucose deprivation contributes to the development of KRAS pathway mutations in tumor cells. Science. 2009;325:1555–9. 10.1126/science.1174229. 19661383 10.1126/science.1174229 PMC2820374

[37] DeNicola GM, Karreth FA, Humpton TJ, Gopinathan A, Wei C, Frese K, et al. Oncogene-induced Nrf2 transcription promotes ROS detoxification and tumorigenesis. Nature. 2011;475:106–9. 10.1038/nature10189. 21734707 10.1038/nature10189 PMC3404470

[38] Mukhopadhyay S, Vander Heiden MG, McCormick F. The metabolic landscape of RAS-driven cancers from biology to therapy. Nat Cancer. 2021;2:271–83. 10.1038/s43018-021-00184-x. 33870211 10.1038/s43018-021-00184-x PMC8045781

[39] Sayin VI, Ibrahim MX, Larsson E, Nilsson JA, Lindahl P, Bergo MO. Antioxidants accelerate lung cancer progression in mice. Sci Transl Med. 2014;6:221ra15 10.1126/scitranslmed.3007653. 24477002 10.1126/scitranslmed.3007653

[40] Shaw AT, Winslow MM, Magendantz M, Ouyang C, Dowdle J, Subramanian A, et al. Selective killing of K-ras mutant cancer cells by small molecule inducers of oxidative stress. Proc Natl Acad Sci USA. 2011;108:8773–8. 10.1073/pnas.1105941108. 21555567 10.1073/pnas.1105941108 PMC3102385

[41] Yang Z, Liang S-Q, Saliakoura M, Yang H, Vassella E, Konstantinidou G, et al. Synergistic effects of FGFR1 and PLK1 inhibitors target a metabolic liability in KRAS-mutant cancer. EMBO Mol Med. 2021;13:e13193. 10.15252/emmm.202013193. 34369083 10.15252/emmm.202013193 PMC8422071

[42] Unda BK, Chalil L, Yoon S, Kilpatrick S, Irwin C, Xing S, et al. Impaired OTUD7A-dependent Ankyrin regulation mediates neuronal dysfunction in mouse and human models of the 15q13.3 microdeletion syndrome. Mol Psychiatry. 2023;28:1747–69. 10.1038/s41380-022-01937-5. 36604605 10.1038/s41380-022-01937-5 PMC10208958

[43] Uddin M, Unda BK, Kwan V, Holzapfel NT, White SH, Chalil L, et al. OTUD7A regulates neurodevelopmental phenotypes in the 15q13.3 microdeletion syndrome. Am J Hum Genet. 2018;102:278–95. 10.1016/j.ajhg.2018.01.006. 29395074 10.1016/j.ajhg.2018.01.006 PMC5985537

[44] Olie CS, O’Brien DP, Jones HBL, Liang Z, Damianou A, Sur-Erdem I, et al. Deubiquitinases in muscle physiology and disorders. Biochem Soc Trans. 2024;52:1085–98. 10.1042/BST20230562. 38716888 10.1042/BST20230562 PMC11346448

[45] Yuan L, Lv Y, Li H, Gao H, Song S, Zhang Y, et al. Deubiquitylase OTUD3 regulates PTEN stability and suppresses tumorigenesis. Nat Cell Biol. 2015;17:1169–81. 10.1038/ncb3218. 26280536 10.1038/ncb3218

[46] Jiang X, Stockwell BR, Conrad M. Ferroptosis: mechanisms, biology, and role in disease. Nat Rev Mol Cell Biol. 2021;22:266–82. 10.1038/s41580-020-00324-8. 33495651 10.1038/s41580-020-00324-8 PMC8142022

[47] Chen X, Kang R, Kroemer G, Tang D. Broadening horizons: the role of ferroptosis in cancer. Nat Rev Clin Oncol. 2021;18:280–96. 10.1038/s41571-020-00462-0. 33514910 10.1038/s41571-020-00462-0

[48] Mou Y, Wang J, Wu J, He D, Zhang C, Duan C, et al. Ferroptosis, a new form of cell death: opportunities and challenges in cancer. J Hematol Oncol. 2019;12:34 10.1186/s13045-019-0720-y. 30925886 10.1186/s13045-019-0720-y PMC6441206

[49] Bruedigam C, Porter AH, Song A, Vroeg In de Wei G, Stoll T, Straube J, et al. Imetelstat-mediated alterations in fatty acid metabolism to induce ferroptosis as a therapeutic strategy for acute myeloid leukemia. Nat Cancer. 2024;5:47–65. 10.1038/s43018-023-00653-5. 37904045 10.1038/s43018-023-00653-5 PMC10824665

[50] Li H, Sun Y, Yao Y, Ke S, Zhang N, Xiong W, et al. USP8-governed GPX4 homeostasis orchestrates ferroptosis and cancer immunotherapy. Proc Natl Acad Sci USA. 2024;121:e2315541121. 10.1073/pnas.2315541121. 38598341 10.1073/pnas.2315541121 PMC11032464

[51] Garcia-Martinez L, Zhang Y, Nakata Y, Chan HL, Morey L. Epigenetic mechanisms in breast cancer therapy and resistance. Nat Commun. 2021;12:1786. 10.1038/s41467-021-22024-3. 33741974 10.1038/s41467-021-22024-3 PMC7979820

[52] Dawson MA, Kouzarides T. Cancer epigenetics: from mechanism to therapy. Cell. 2012;150:12–27. 10.1016/j.cell.2012.06.013. 22770212 10.1016/j.cell.2012.06.013

[53] Paredes F, Williams HC, San Martin A. Metabolic adaptation in hypoxia and cancer. Cancer Lett. 2021;502:133–42. 10.1016/j.canlet.2020.12.020. 33444690 10.1016/j.canlet.2020.12.020 PMC8158653

[54] Zhang Y, Wu M-J, Lu W-C, Li Y-C, Chang CJ, Yang J-Y. Metabolic switch regulates lineage plasticity and induces synthetic lethality in triple-negative breast cancer. Cell Metab. 2024;36:193–.e8. 10.1016/j.cmet.2023.12.003. 38171333 10.1016/j.cmet.2023.12.003

[55] Huang J, Yin Q, Wang Y, Zhou X, Guo Y, Tang Y, et al. EZH2 inhibition enhances PD-L1 protein stability through USP22-mediated deubiquitination in colorectal cancer. Adv Sci. 2024;11:e2308045. 10.1002/advs.202308045. 10.1002/advs.202308045 PMC11187912 38520088

[56] Yang J, Xu J, Wang W, Zhang B, Yu X, Shi S. Epigenetic regulation in the tumor microenvironment: molecular mechanisms and therapeutic targets. Signal Transduct Target Ther. 2023;8:210 10.1038/s41392-023-01480-x. 37217462 10.1038/s41392-023-01480-x PMC10203321

[57] Lim S-O, Li C-W, Xia W, Cha J-H, Chan L-C, Wu Y, et al. Deubiquitination and stabilization of PD-L1 by CSN5. Cancer Cell. 2016;30:925–39. 10.1016/j.ccell.2016.10.010. 27866850 10.1016/j.ccell.2016.10.010 PMC5171205

[58] Chen Z, Han F, Du Y, Shi H, Zhou W. Hypoxic microenvironment in cancer: molecular mechanisms and therapeutic interventions. Signal Transduct Target Ther. 2023;8:70 10.1038/s41392-023-01332-8. 36797231 10.1038/s41392-023-01332-8 PMC9935926

[59] Chen S, Liao C, Hu H, Liao J, Chen Z, Li S, et al. Hypoxia-driven tumor stromal remodeling and immunosuppressive microenvironment in scirrhous HCC. Hepatology. 2024;79:780–97. 10.1097/HEP.0000000000000599. 37725755 10.1097/HEP.0000000000000599

[60] Bai R, Li Y, Jian L, Yang Y, Zhao L, Wei M. The hypoxia-driven crosstalk between tumor and tumor-associated macrophages: mechanisms and clinical treatment strategies. Mol Cancer. 2022;21:177. 10.1186/s12943-022-01645-2. 36071472 10.1186/s12943-022-01645-2 PMC9454207

[61] Denk D, Greten FR. Inflammation: the incubator of the tumor microenvironment. Trends Cancer. 2022;8:901–14. 10.1016/j.trecan.2022.07.002. 35907753 10.1016/j.trecan.2022.07.002

