# Supplementary material for: Suppressing the OTUD7A/KDM5B/GABPA axis enhances the sensitivity of cisplatin through inducing ferroptosis in KRAS-mutant LUAD
Source: Cell Death Dis. 2025 Dec 20;17(1):112. doi: 10.1038/s41419-025-08337-x (PMC12848020; doi:10.1038/s41419-025-08337-x)
Supplement: Supplementary file 1 — supplementary material [file 41419_2025_8337_MOESM1_ESM.docx]

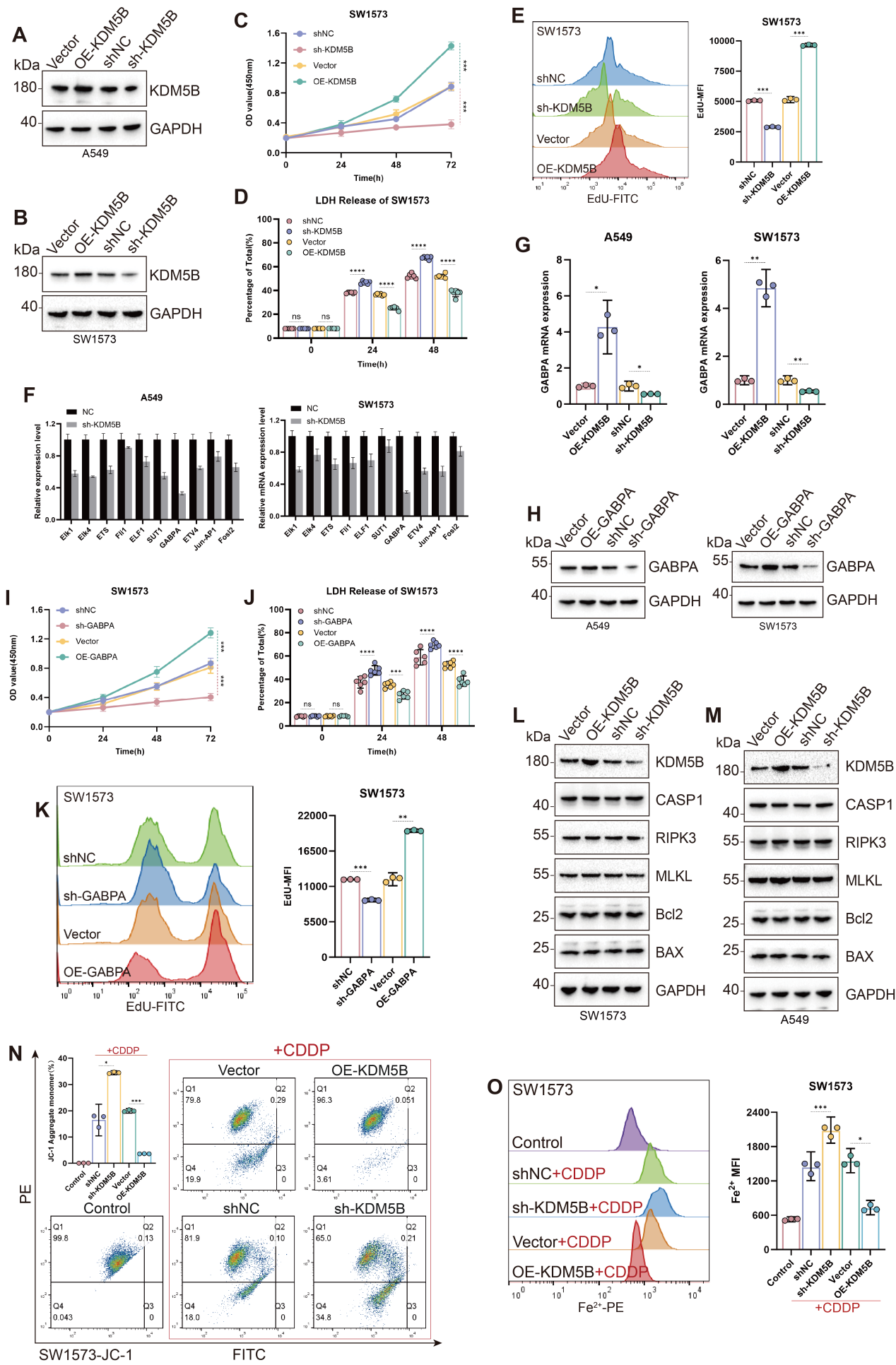


**Supplementary Figure 1:**

**A-B:** The expression levels of KDM5B in stable transfectants of A549 and SW1573 cells (Vector, OE-KDM5B, shNC, sh-KDM5B). **C-D:** The viability and mortality of SW1573 cells were assessed at 24, 48, and 72 hours following stable knockdown and overexpression of KDM5B using CCK-8 and LDH assays. **E:** The proliferation rate of SW1573 cells was measured using the EdU assay following stable knockdown and overexpression of KDM5B. **F:** RT-qPCR validation of changes in the mRNA expression levels of the top 10 genes reported by ChIP-Seq in A549 and SW1573 cells following normal and KDM5B knockdown. **G**: RT-qPCR was utilized to validate the changes in GABPA mRNA expression levels following KDM5B knockdown and overexpression in A549 and SW1573 cells. **H:** The expression levels of GABPA in stable transfectants of A549 and SW1573 cells (Vector, OE-GABPA, shNC, sh-GABPA). **I-J**: The viability and mortality of SW1573 cells were assessed at 24, 48, and 72 hours following stable knockdown and overexpression of GABPA using CCK-8 and LDH assays. **K:** The proliferation rate of SW1573 cells was measured using the EdU assay following stable knockdown and overexpression of GABPA. **L-M:** WB analysis detects key markers of programmed cell death, necroptosis, and pyroptosis across two LUAD cell lines, SW1573 and A549. **N**: The changes in mitochondrial membrane potential of SW1573 cells were assessed using the JC-1 probe after stable knockdown and overexpression of KDM5B. (All groups except the Control were pre-treated with 265 μM cisplatin for 10 hours, with O undergoing the same treatment). **O:** In SW1573 cells, changes in intracellular Fe²⁺ levels were detected using an iron ion fluorescent probe. All experimental procedures were independently replicated three times, demonstrating consistent outcomes. Error bars represent the mean ± S.D. *P < 0.05, **P < 0.01, ***P < 0.001, ****P < 0.0001.


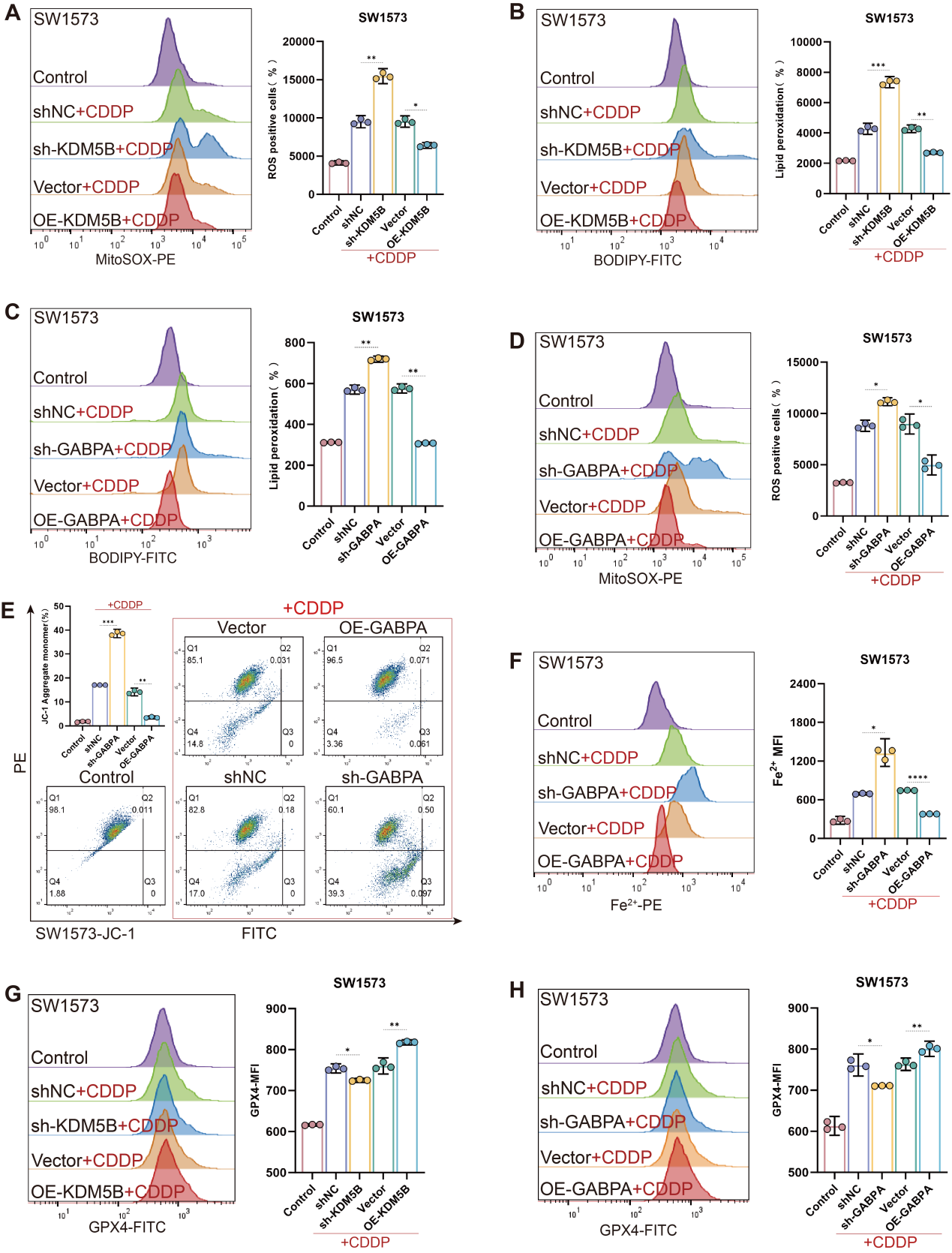


**Supplementary Figure 2:**

**A-B**: The generation of ROS and lipid ROS in SW1573 cells was detected using MitoSOX and BODIPY C11 probes following stable knockdown and overexpression of KDM5B. (All groups except the Ctrl were pre-treated with 265 μM cisplatin for 10 hours, with B-H undergoing the same treatment). **C-D**: The production of ROS and lipid ROS in SW1573 cells was detected using MitoSOX and BODIPY C11 probes following stable knockdown and overexpression of GABPA. **E**: The changes in mitochondrial membrane potential of SW1573 cells were assessed using the JC-1 probe after stable knockdown and overexpression of GABPA. **F:** In SW1573 cells, changes in intracellular Fe²⁺ levels were detected using an iron ion fluorescent probe. **G**: Flow cytometry was employed to evaluate changes in GPX4 expression levels in SW1573 cells after stable knockdown and overexpression of KDM5B. **H**: Flow cytometry was employed to evaluate changes in GPX4 expression levels in SW1573 cells after stable knockdown and overexpression of GABPA. All experimental procedures were independently replicated three times, demonstrating consistent outcomes. Error bars represent the mean ± S.D. *P < 0.05, **P < 0.01, ***P < 0.001, ****P < 0.0001.


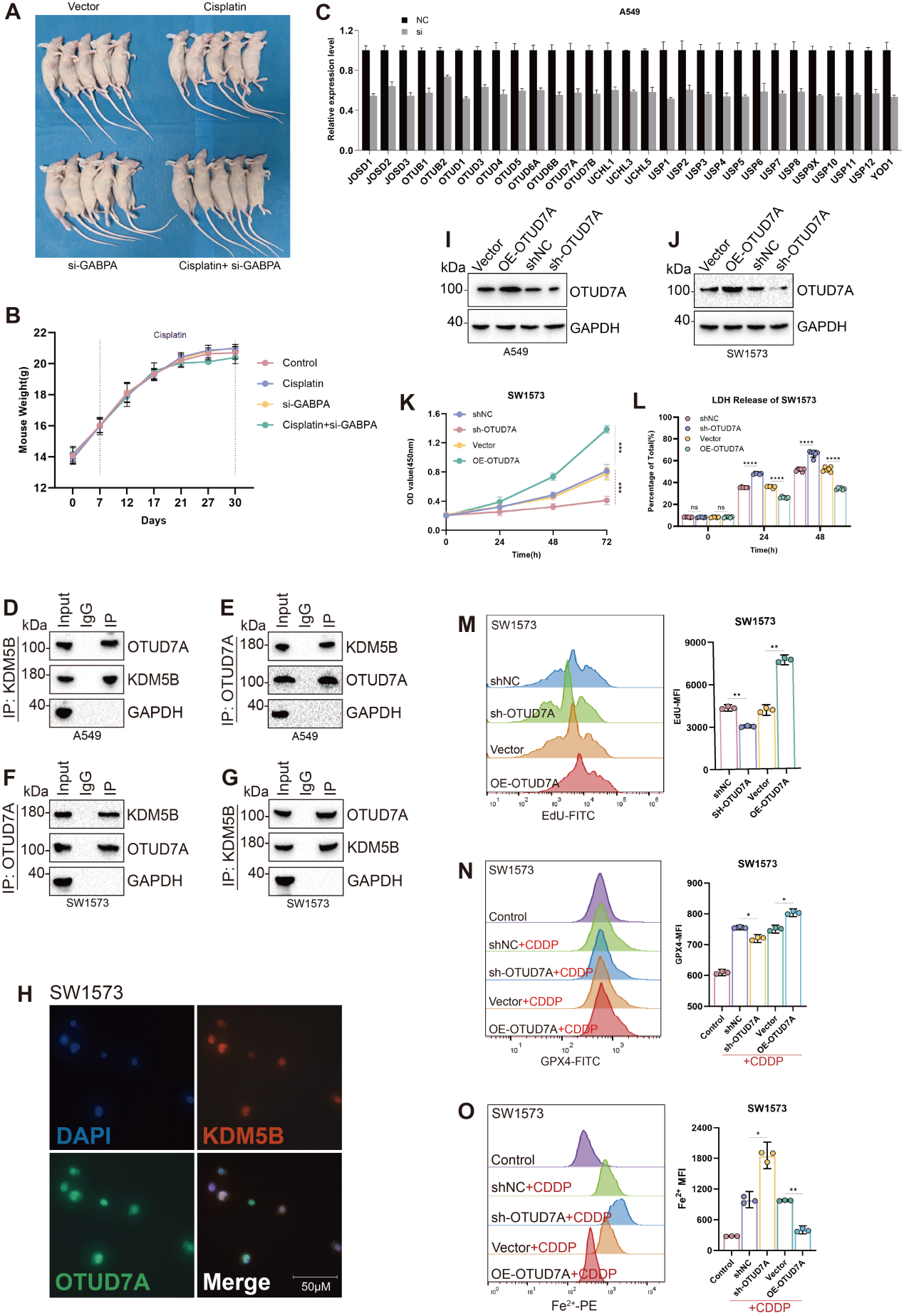


**Supplementary Figure 3:**

**A-B**: Growth conditions and body weight curves of nude mice following subcutaneous xenografts of human lung-derived organoid models. **C**: RT-qPCR validation of the transfection efficiency of 30 siRNAs transiently transfected into A549 cells. **D-G**: Co-immunoprecipitation experiments conducted in A549 and SW1573 cells to verify the interaction between KDM5B and OTUD7A. **H**: Immunofluorescence co-localization experiments demonstrating co-localization of KDM5B and OTUD7A in SW1573 cells. **I-J**: The expression levels of OTUD7A in stable transfectants of A549 and SW1573 cells (Vector, OE-OTUD7A, shNC, sh-OTUD7A). **K-L**: The viability and mortality of SW1573 cells were assessed at 24, 48, and 72 hours following stable knockdown and overexpression of OTUD7A using CCK-8 and LDH assays. **M:** The proliferation rate of SW1573 cells was measured using the EdU assay following stable knockdown and overexpression of OTUD7A. **N**: Flow cytometry was employed to assess changes in GPX4 expression levels within SW1573 cells following stable knockdown and overexpression of OTUD7A (All groups except the Control were pre-treated with 265 μM cisplatin for 10 hours, with O undergoing the same treatment). **O:** In SW1573 cells, changes in intracellular Fe²⁺ levels were detected using an iron ion fluorescent probe. Error bars represent the mean ± S.D. *P < 0.05, **P < 0.01, ***P < 0.001, ****P < 0.0001.


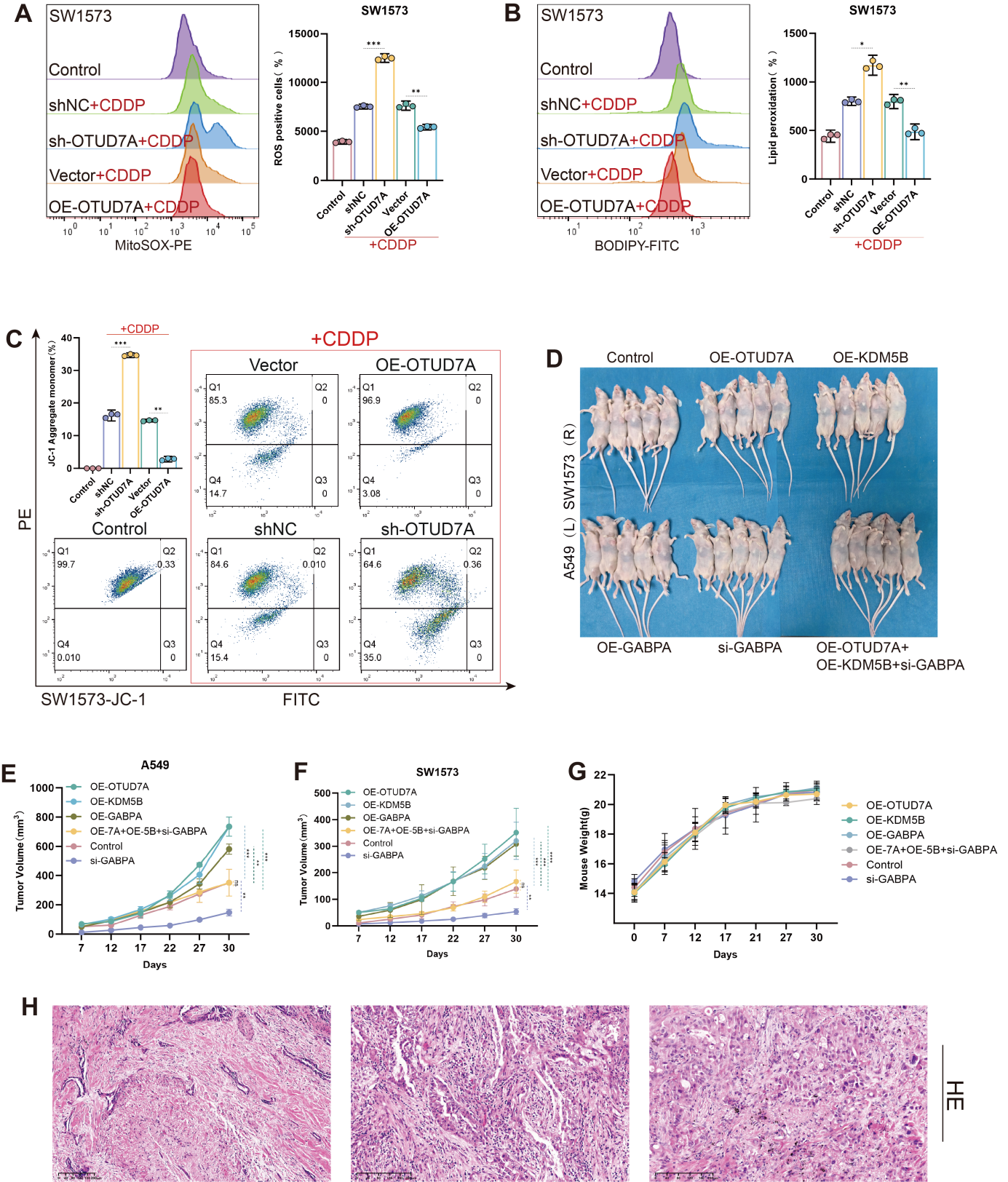


**Supplementary Figure 4:**

**A-B**: Changes in intracellular ROS and lipid ROS production in SW1573 cells following stable knockdown and overexpression of OTUD7A, detected using MitoSOX and BODIPY C11 probes. (All groups except the Control were pre-treated with 265 μM cisplatin for 10 hours, with C undergoing the same treatment). **C**: The alterations in mitochondrial membrane potential in SW1573 cells were assessed using the JC-1 probe after stable knockdown and overexpression of OTUD7A. **D-G**: Subcutaneous tumor models were established using A549 and SW1573 cell lines in nude mice, with E-F presenting the tumor volume growth curves for A549 and SW1573, and G showing the body weight growth curve of the nude mice. **H:** Immunohistochemical staining for HE was performed on tissues from KRAS-mutant LUAD patients. All experimental procedures were independently replicated three times, demonstrating consistent outcomes. Error bars represent the mean ± S.D. *P < 0.05, **P < 0.01, ***P < 0.001, ****P < 0.0001.


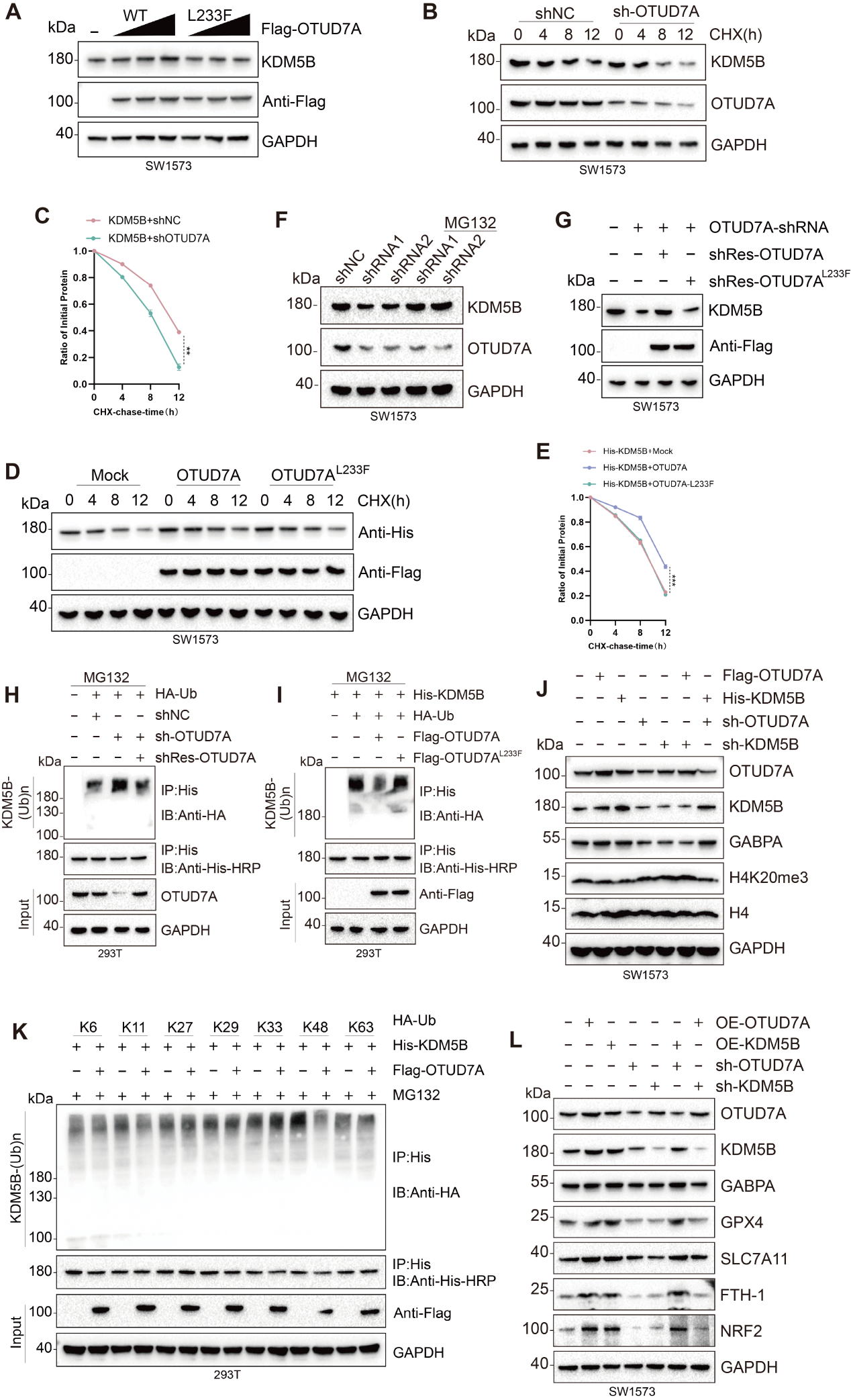


**Supplementary Figure 5**:

**A**: Changes in KDM5B expression levels following gradient transfection of normal and inactive OTUD7A in SW1573 cells. **B-E**: Half-life experiments conducted in SW1573 cells, observing the temporal variations in KDM5B expression levels after the knockdown and overexpression of OTUD7A, as well as the overexpression of inactive OTUD7A, following the addition of actinomycin D. **F**: Detection of KDM5B expression levels via WB in SW1573 cells upon knockdown of OTUD7A in conjunction with the proteasome inhibitor MG132. **G**: Analysis of KDM5B expression levels in SW1573 cells using WB post-knockdown of OTUD7A and concurrent transfection with wild-type or inactive OTUD7A plasmids. **H-I**: In vivo ubiquitination assays performed in 293T cells, examining the ubiquitination levels of the KDM5B protein following knockdown, overexpression, and overexpression of inactive OTUD7A, and simultaneous transfection with wild-type rescue plasmids. **J**: Assessment of the impact of the OTUD7A-KDM5B-GABPA axis on histone modification at the H4K20me3 site in SW1573 cells via WB. **K**: In vivo ubiquitination experiments in 293T cells to observe the changes in ubiquitination sites during the interaction between OTUD7A and KDM5B. **L:** Evaluation of the effect of the OTUD7A-KDM5B-GABPA axis on the expression levels of GPX4, SLC7A11, FTH-1 and NRF2 proteins in SW1573 cells through WB. Error bars represent the mean ± S.D. *P < 0.05, **P < 0.01, ***P < 0.001, ****P < 0.0001.
