# Supplementary figures and images for: Suppressing the OTUD7A/KDM5B/GABPA axis enhances the sensitivity of cisplatin through inducing ferroptosis in KRAS-mutant LUAD
Source: Cell Death Dis. 2025 Dec 20;17(1):112. doi: 10.1038/s41419-025-08337-x (PMC12848020; doi:10.1038/s41419-025-08337-x)

**WB**

**
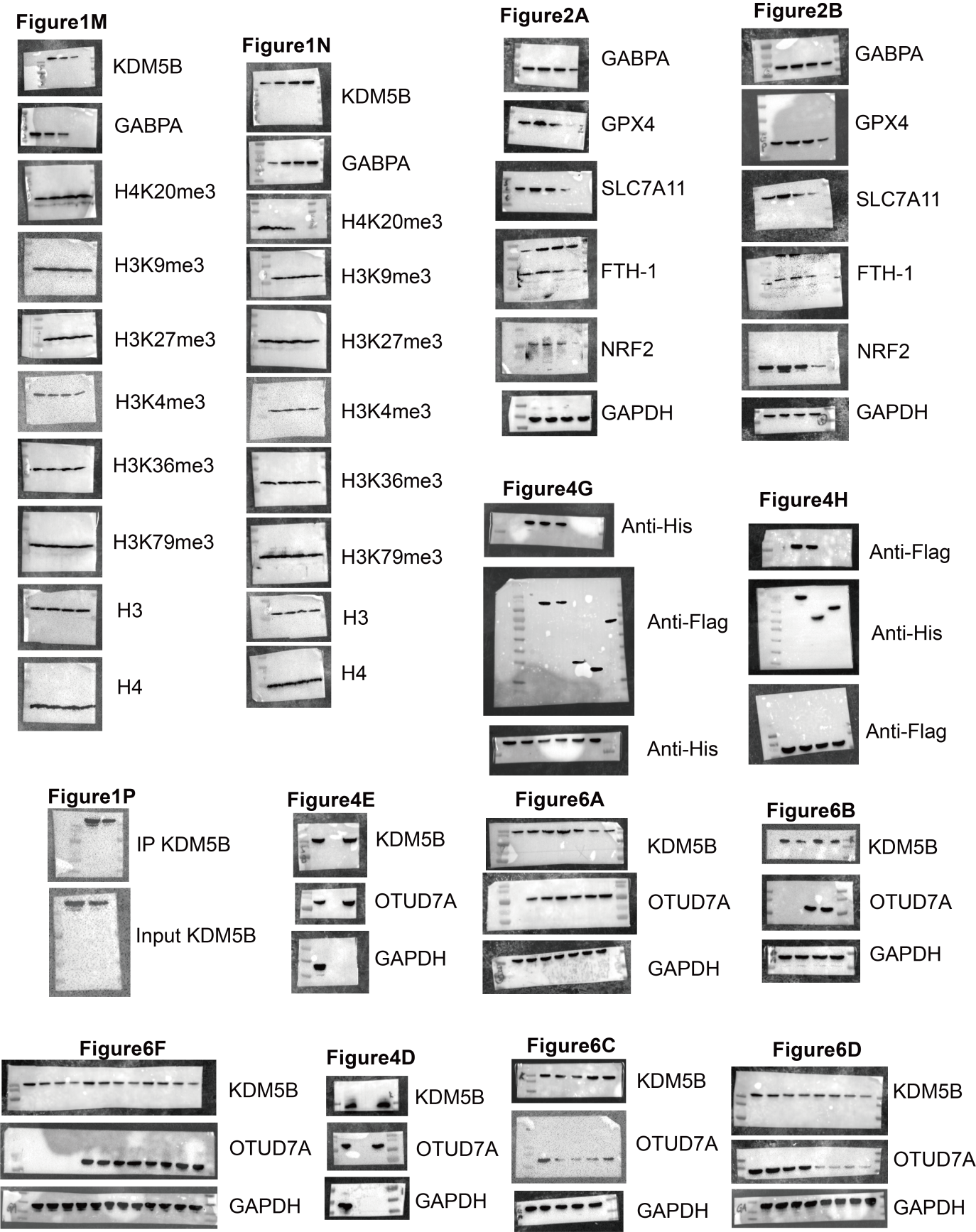
**

**
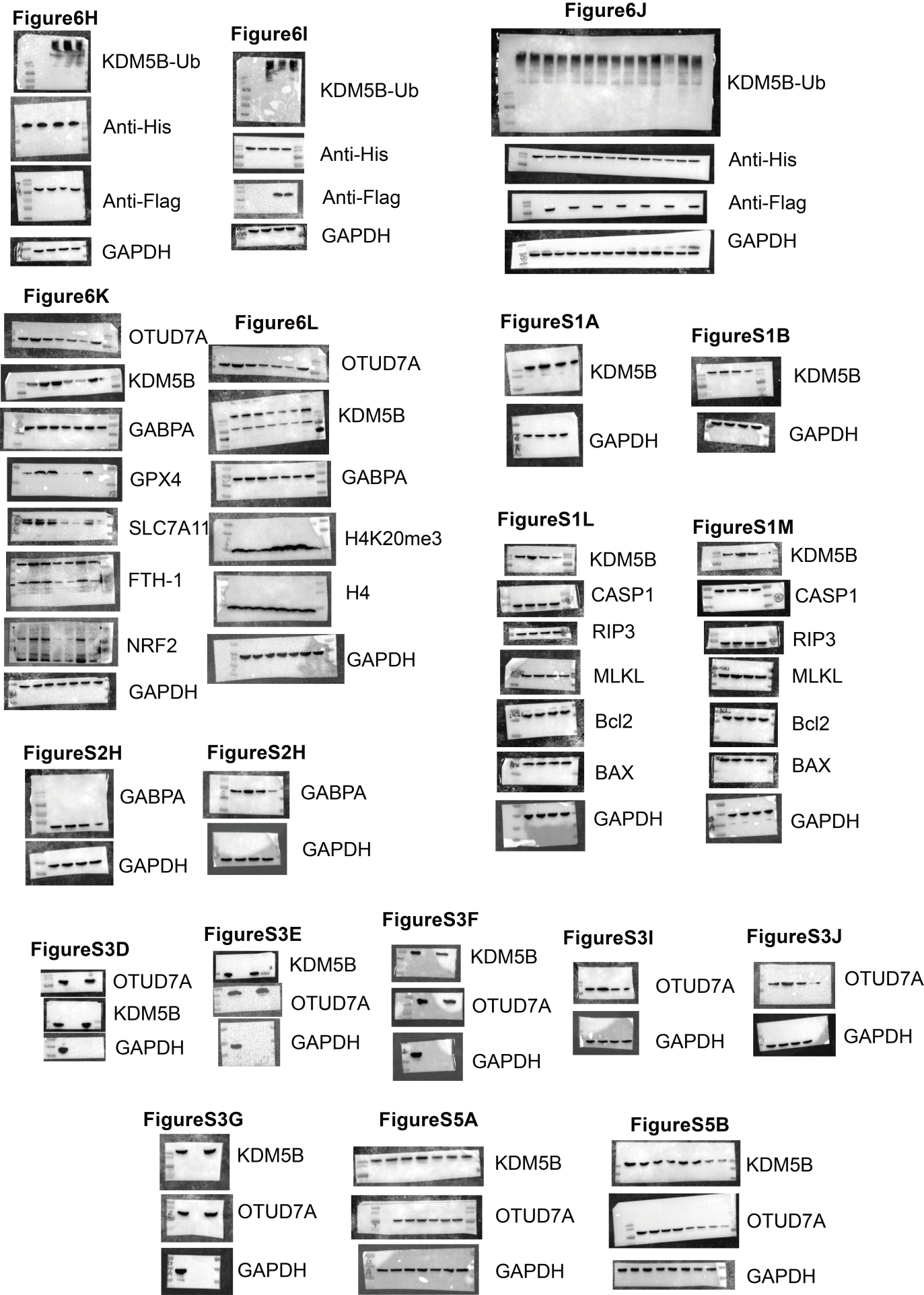
**

**
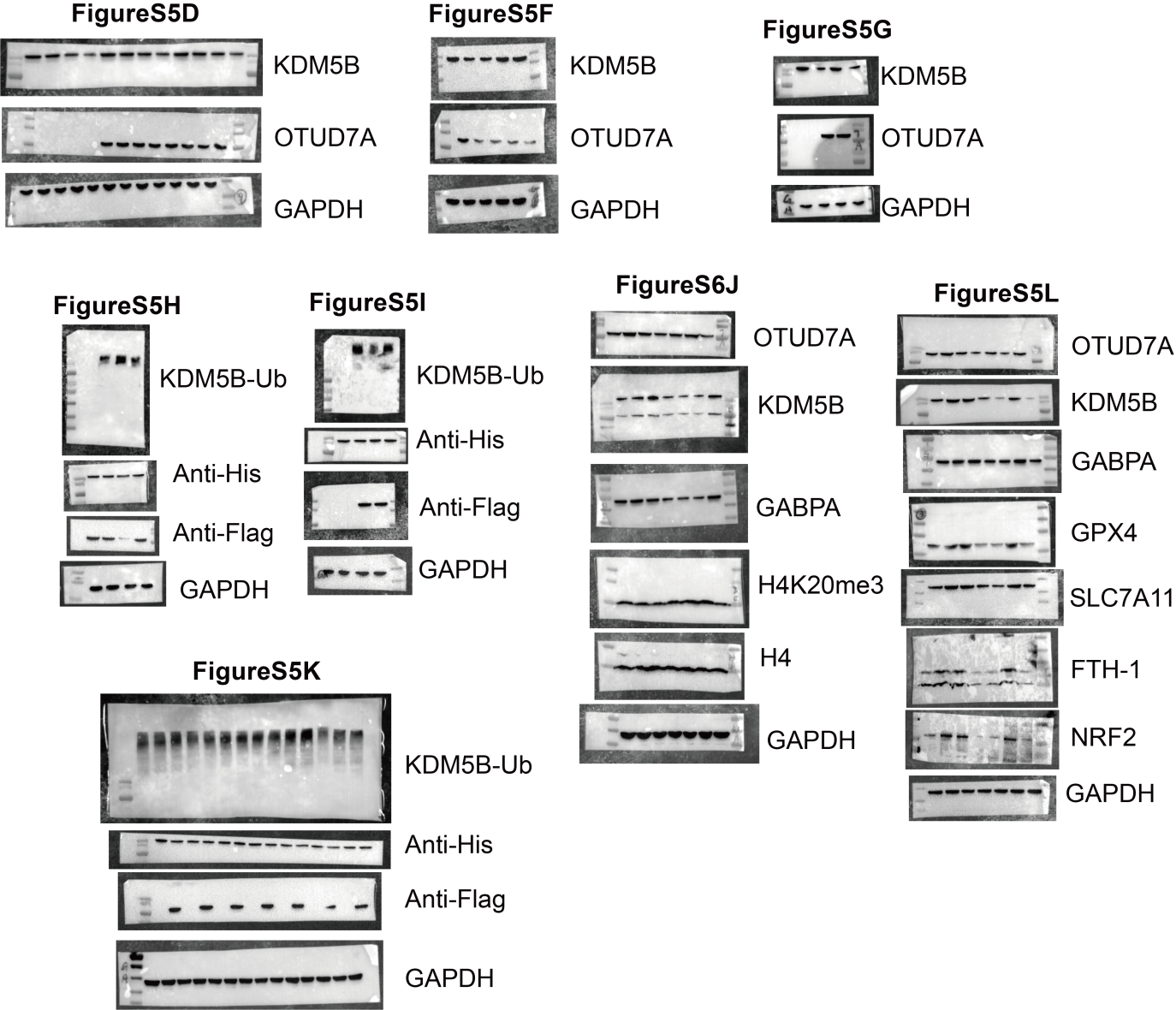
**


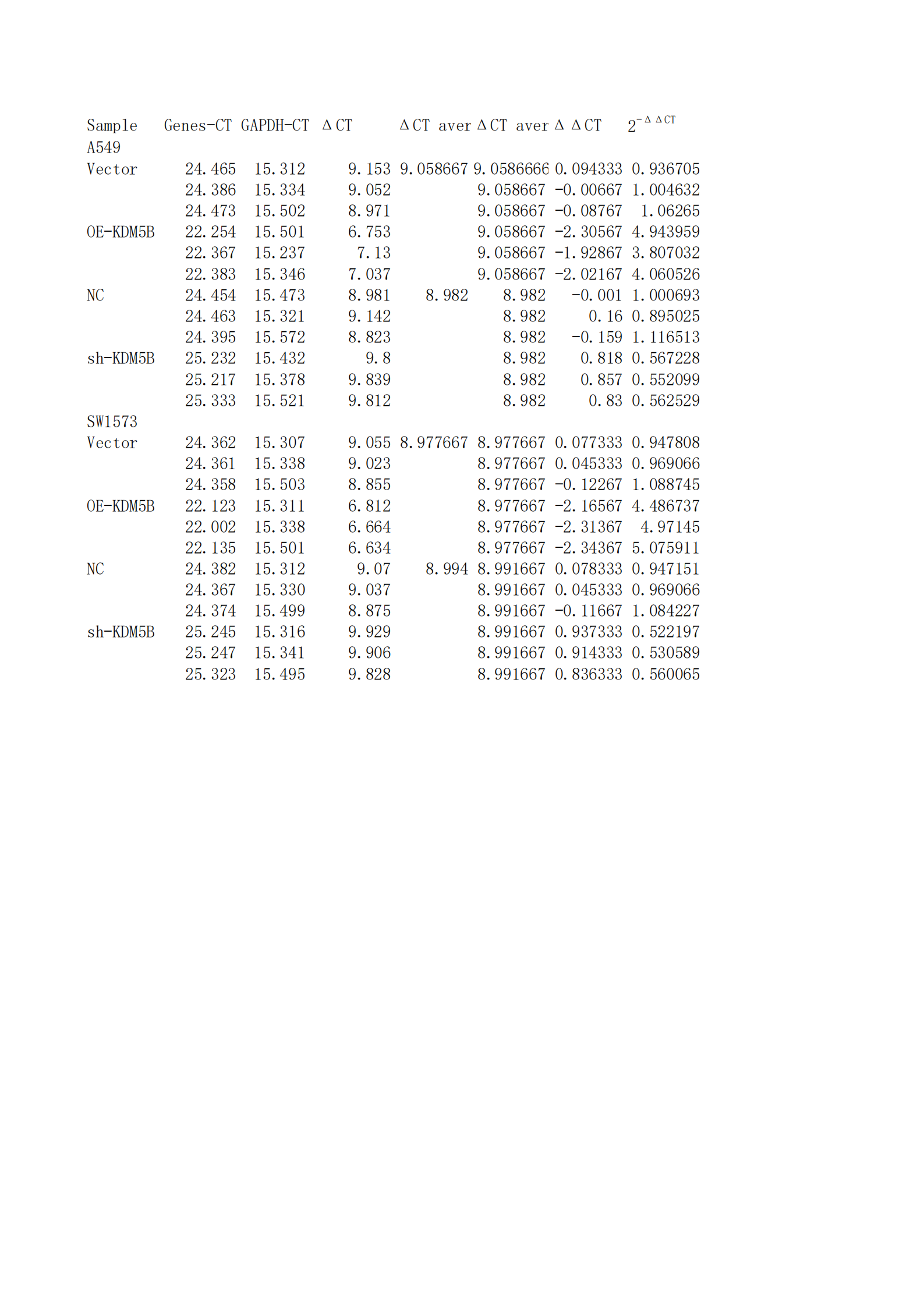

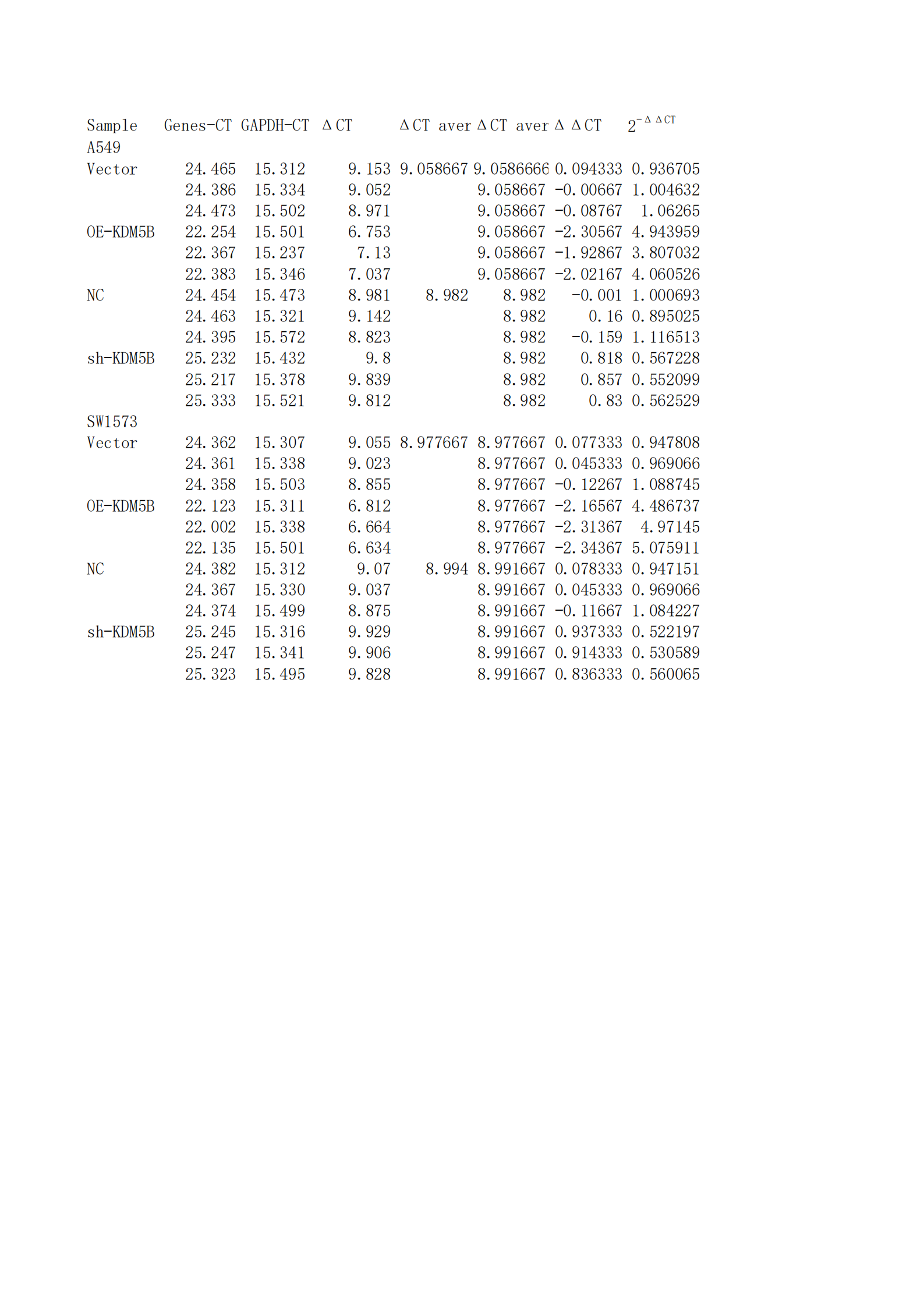

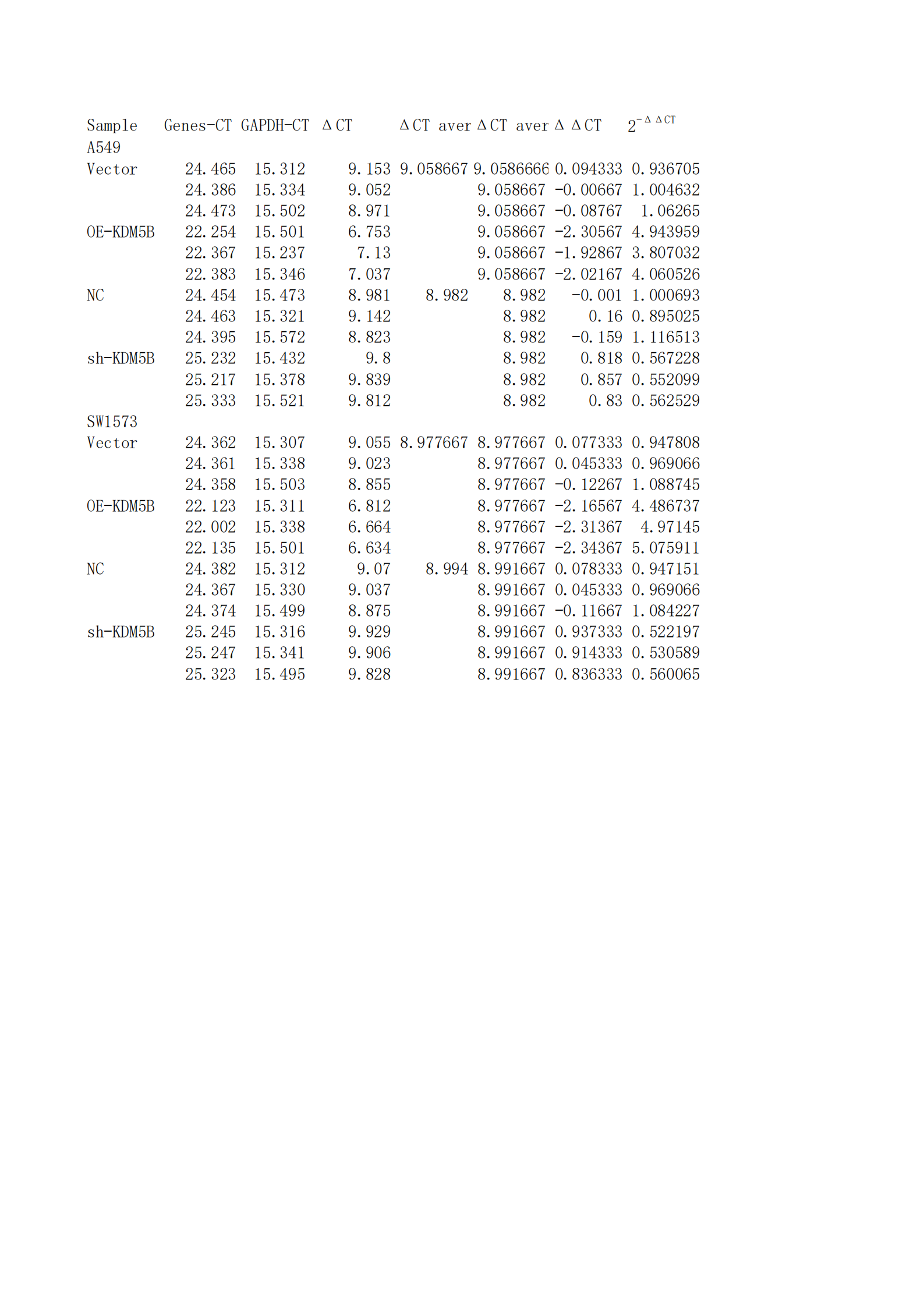

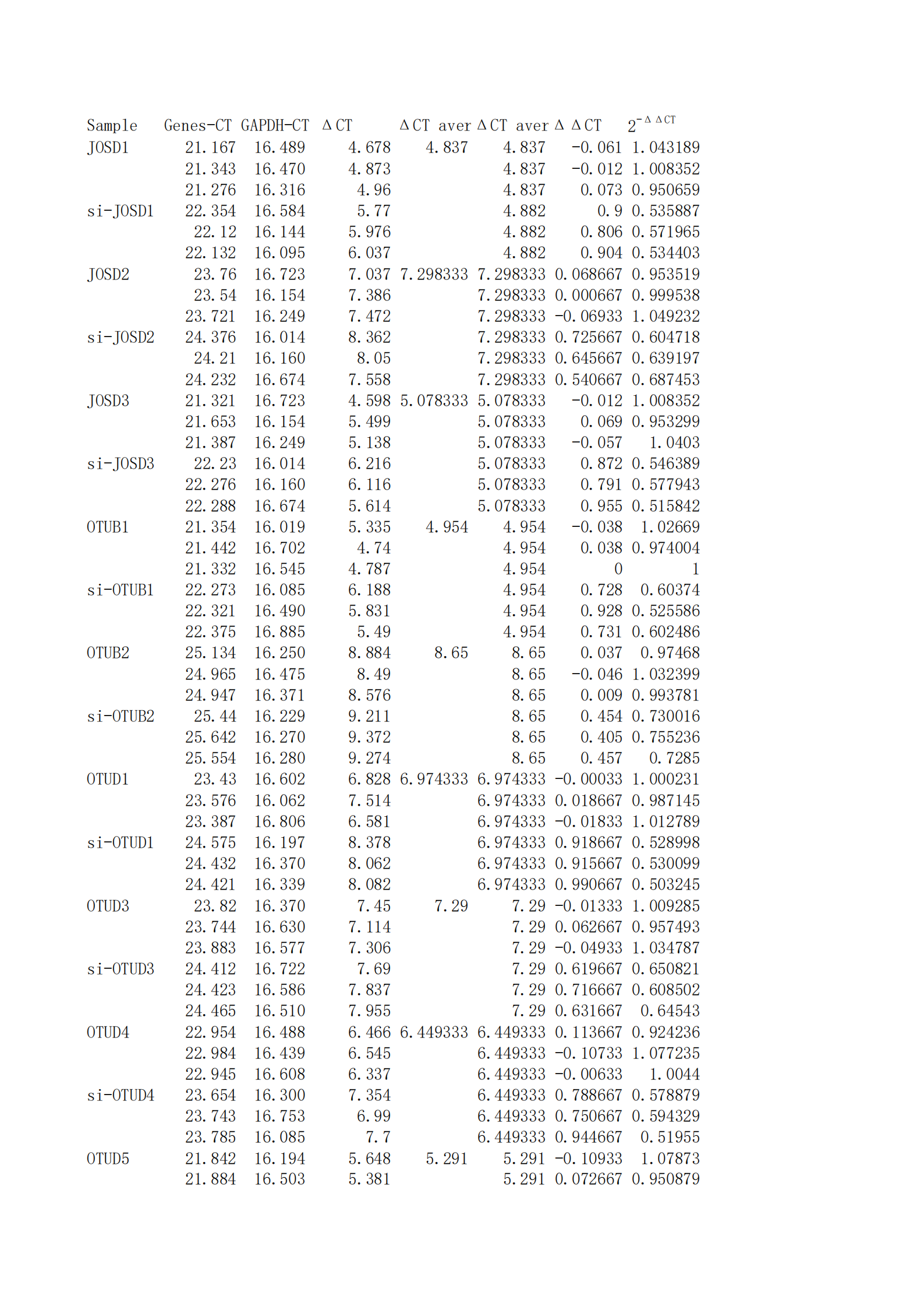


**qPCR**


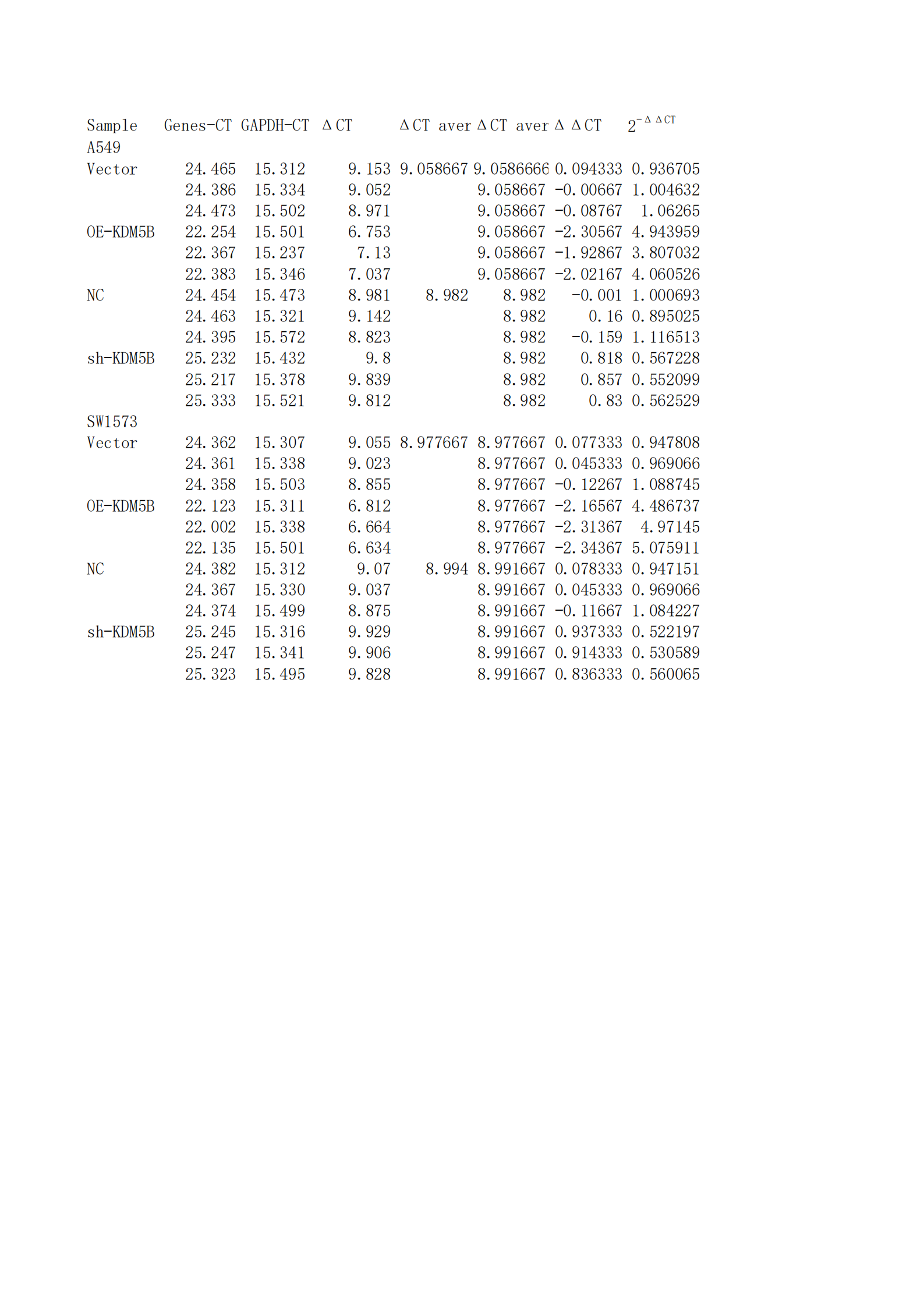


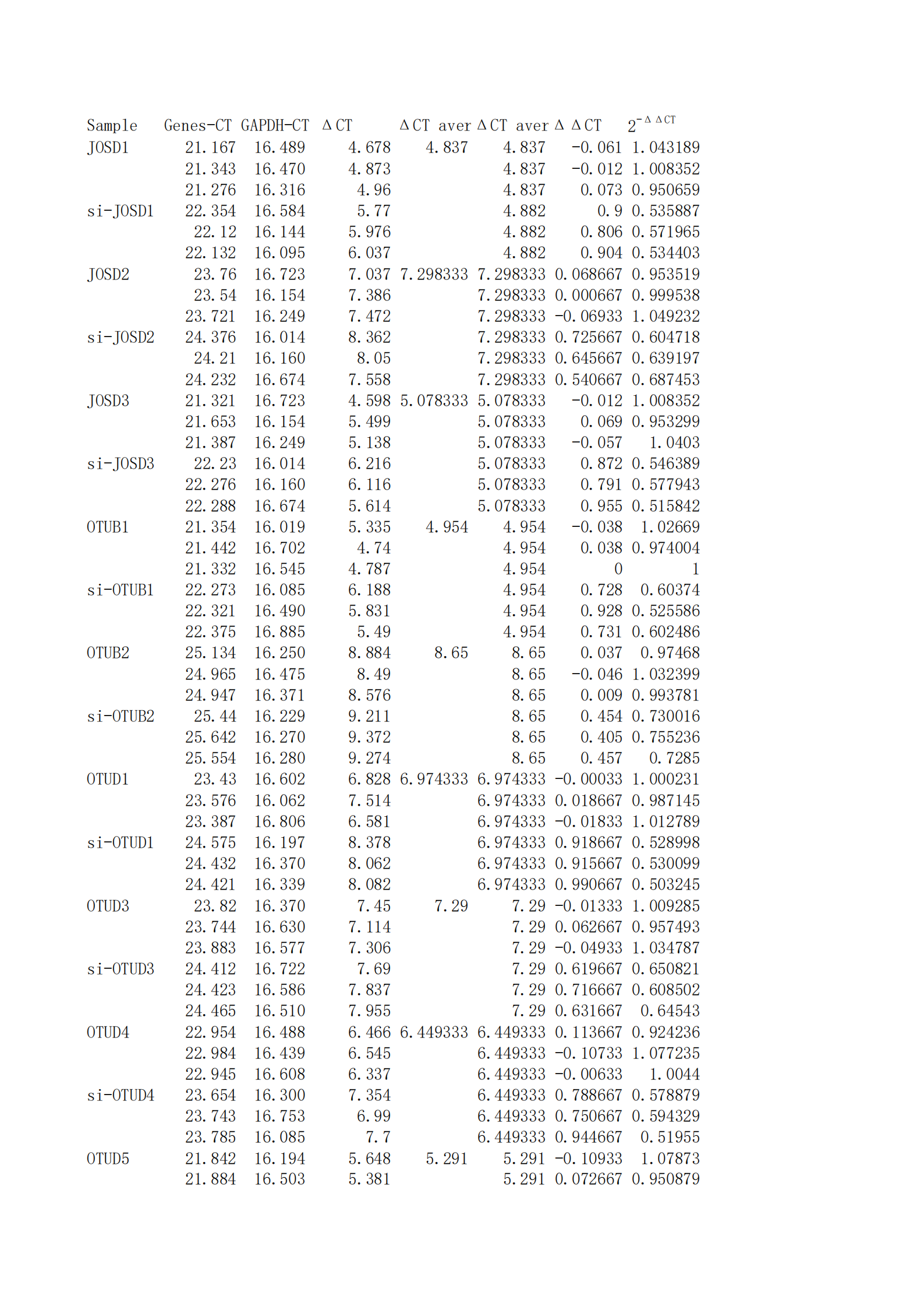


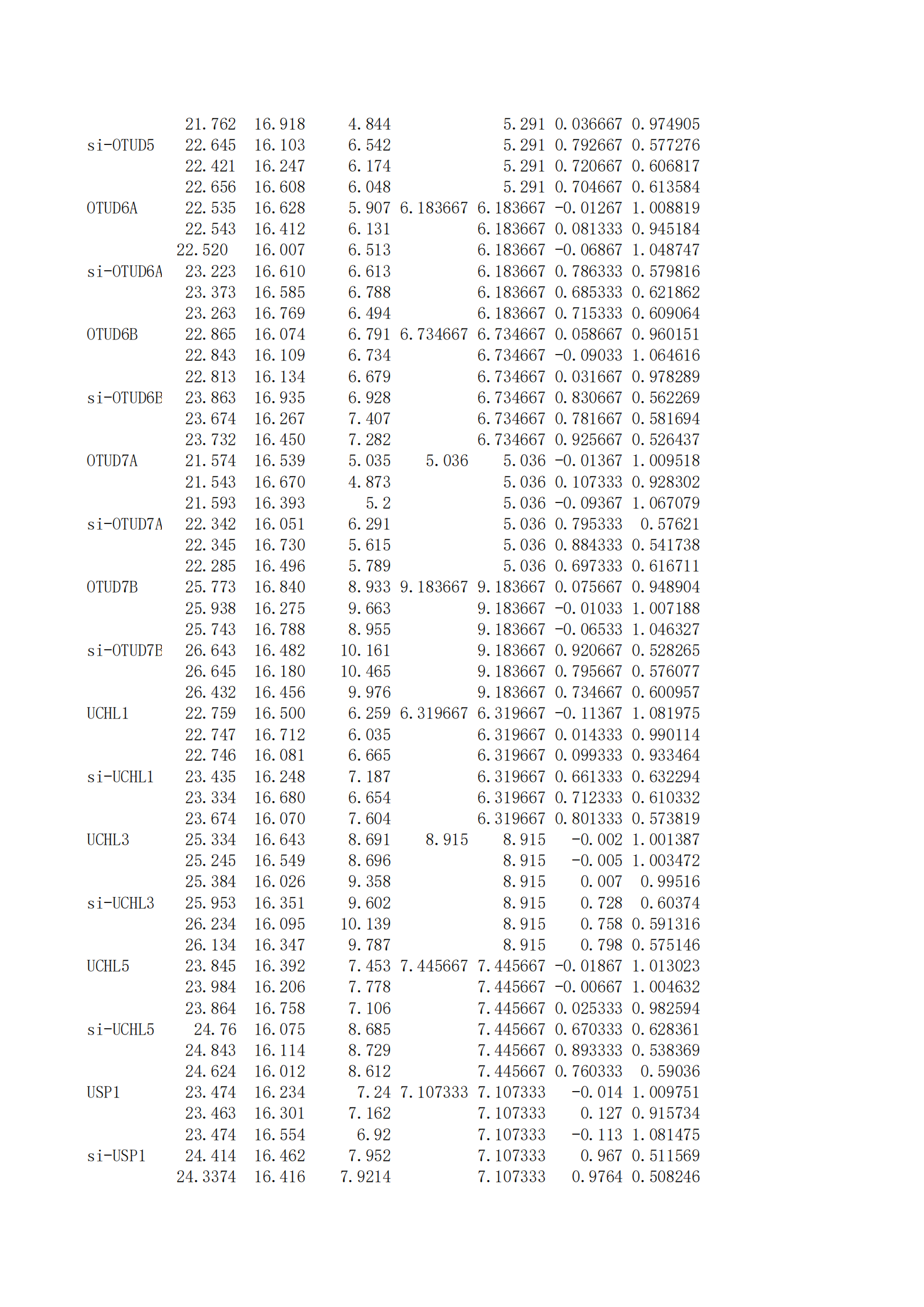


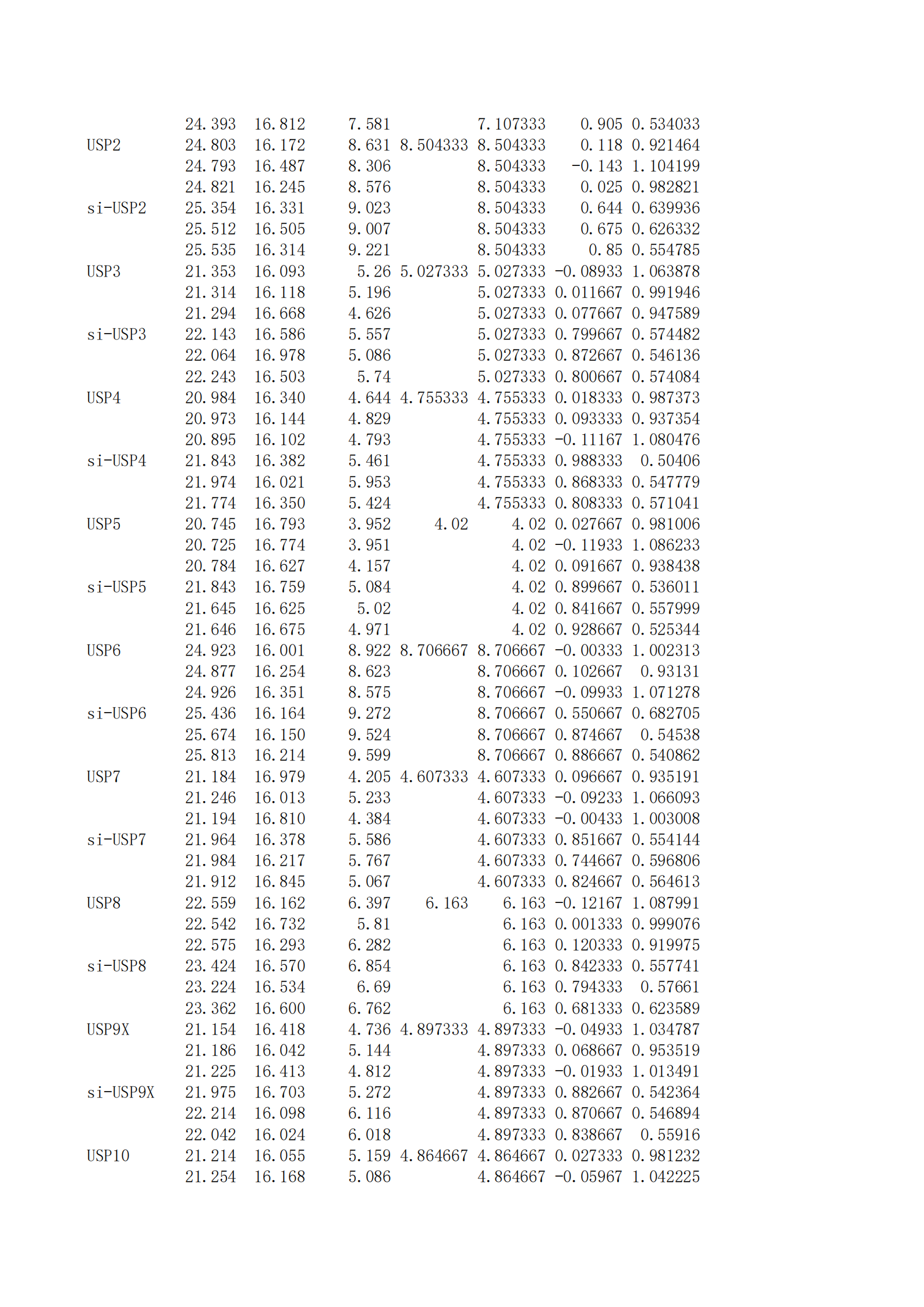


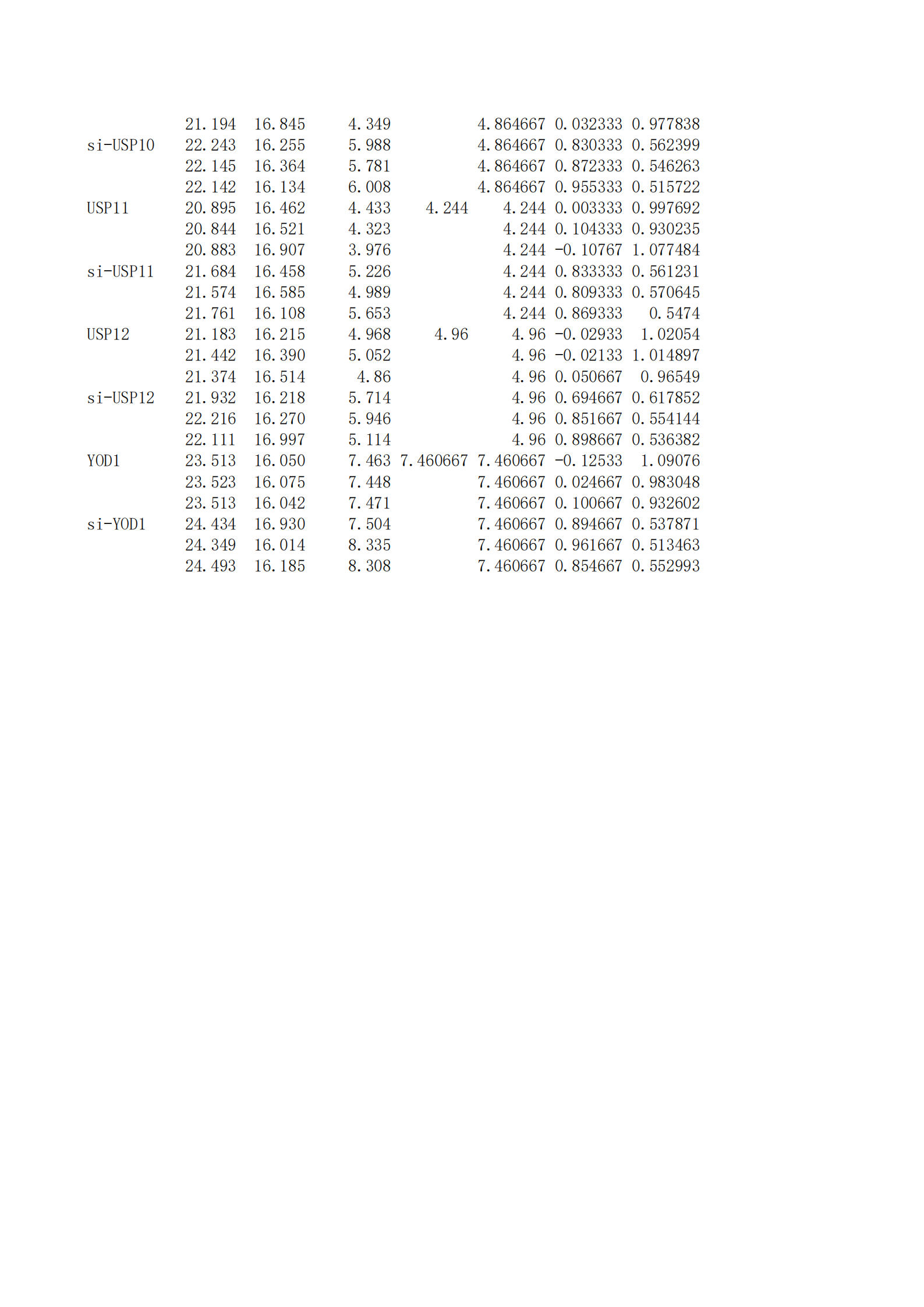


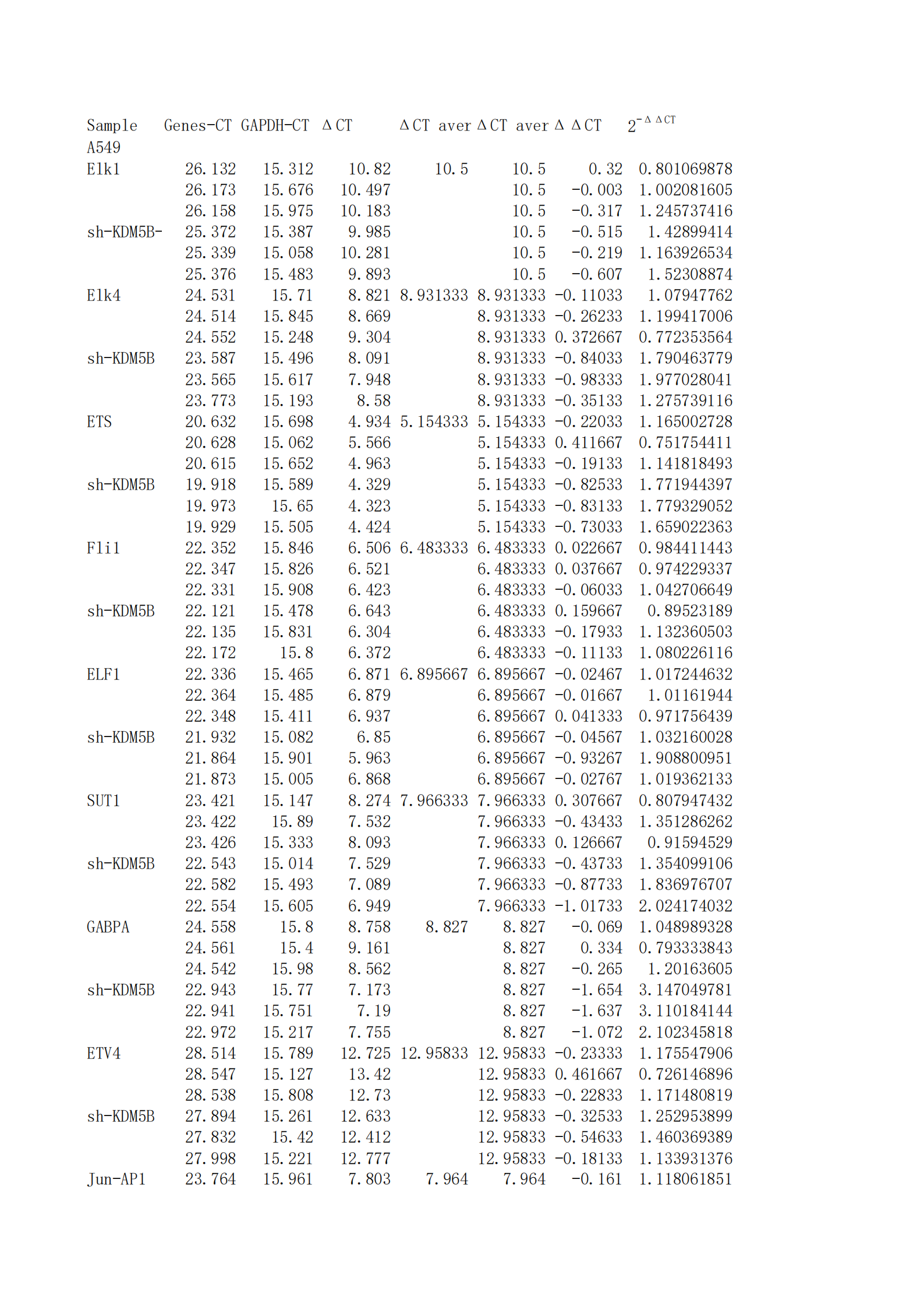


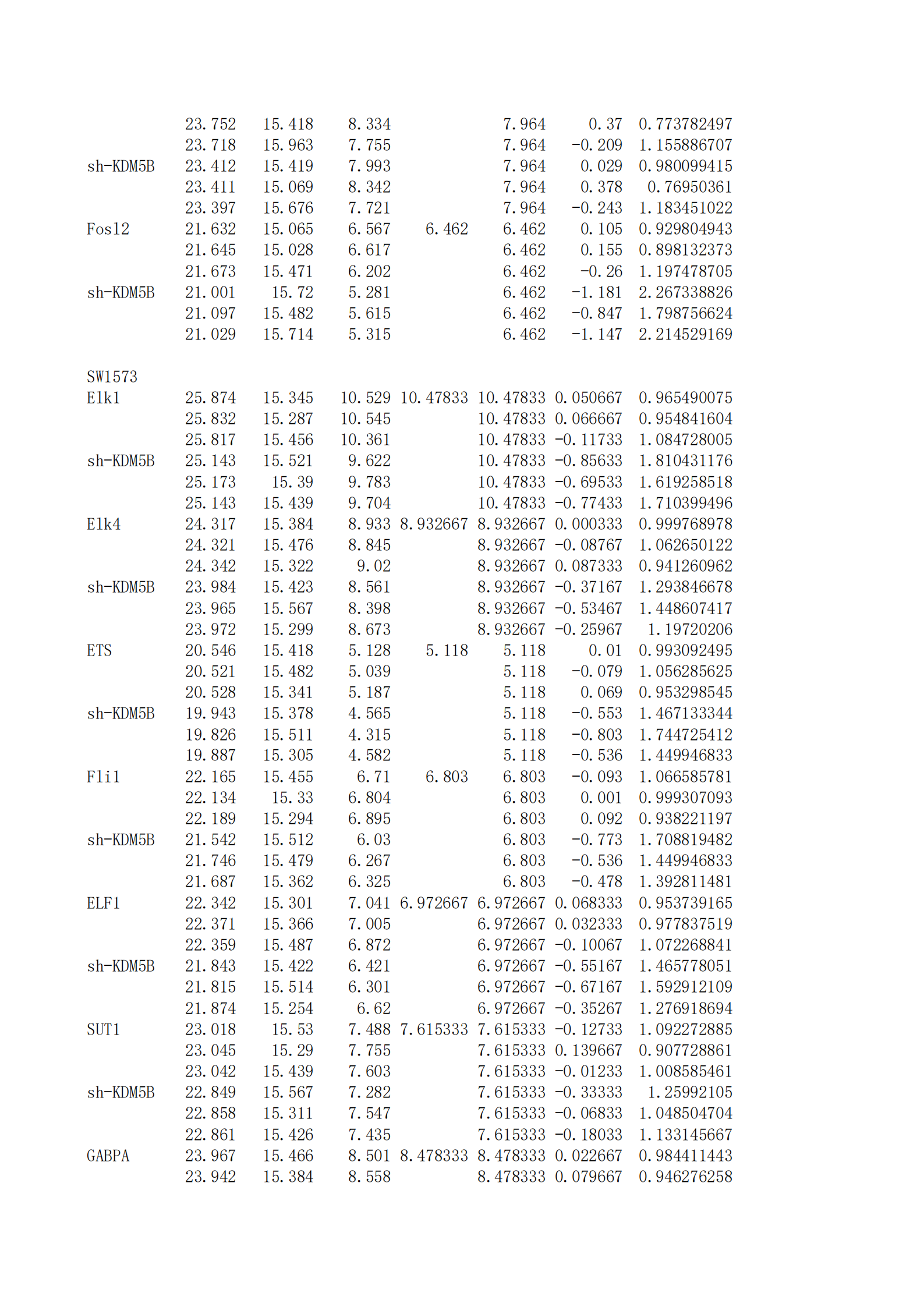


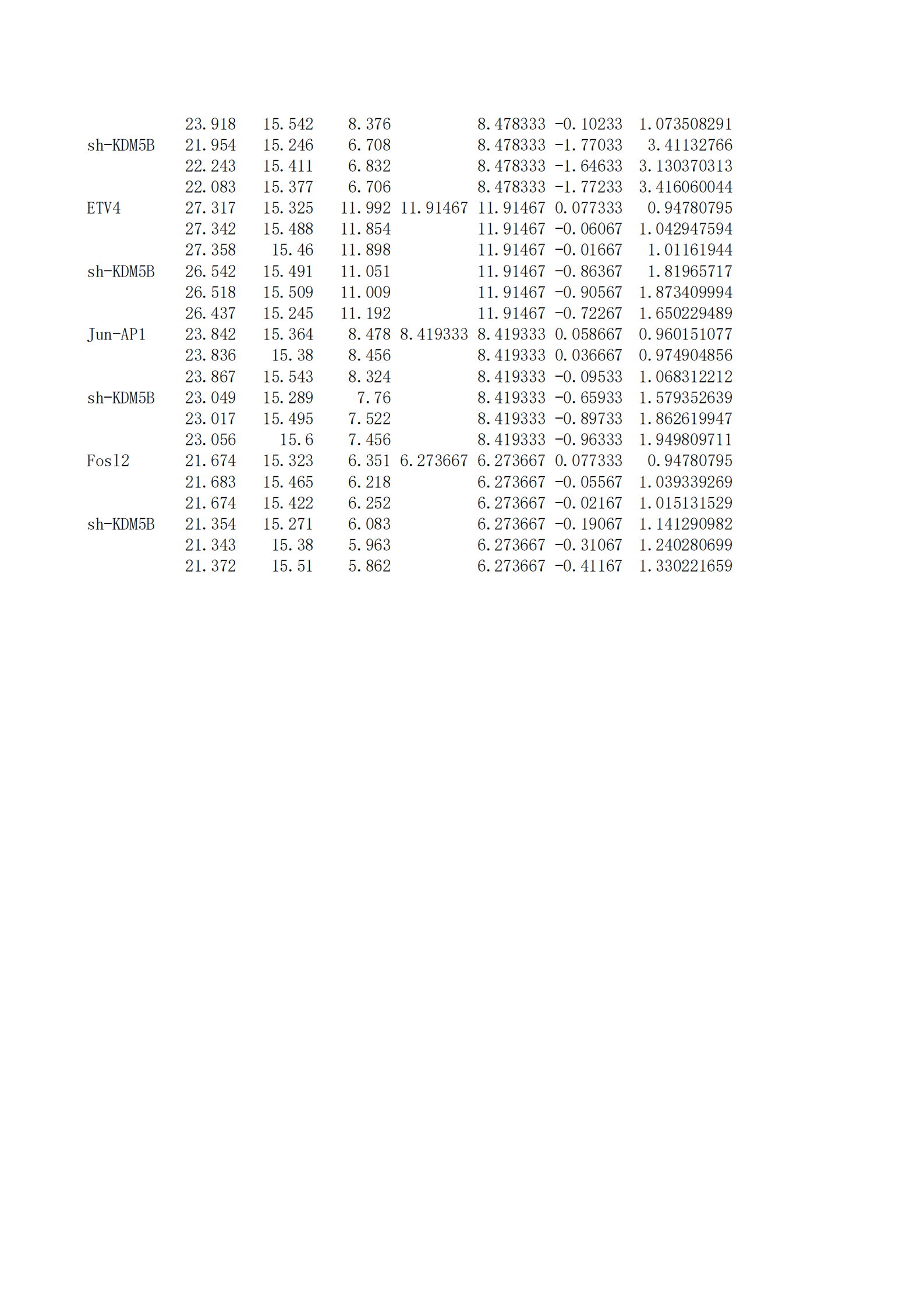

Supplement: Supplementary file 2 — original data [file 41419_2025_8337_MOESM2_ESM.docx]
